# New Insight into the Measurements of Particle-Bound Metals in the Urban and Remote Atmospheres of the Sarajevo Canton and Modeled Impacts of Particulate Air Pollution in Bosnia and Herzegovina

**DOI:** 10.1021/acs.est.1c07037

**Published:** 2022-03-02

**Authors:** Sabina Žero, Silva Žužul, Jasna Huremović, Gordana Pehnec, Ivan Bešlić, Jasmina Rinkovec, Ranka Godec, Noah Kittner, Karla Pavlović, Nino Požar, Juan J. Castillo, Sergio Sanchez, Manousos I. Manousakas, Markus Furger, Andre S.H. Prevot, Griša Močnik, Katja Džepina

**Affiliations:** a Department of Chemistry, Faculty of Science, University of Sarajevo, 71000, Sarajevo, Bosnia and Herzegovina; b Environmental Hygiene Unit, Institute for Medical Research and Occupational Health, 10000, Zagreb, Croatia; c Department of Environmental Sciences and Engineering, University of North Carolina at Chapel Hill, Chapel Hill, North Carolina 27599-7400, United States; d Department of Biotechnology, University of Rijeka, 51000, Rijeka, Croatia; e Clean Air Institute, Washington, DC 20005, United States; f Laboratory of Atmospheric Chemistry, Paul Scherrer Institute, 5232, Villigen, PSI, Switzerland; g Center for Atmospheric Research, University of Nova Gorica, SI-5270, Ajdovščina, Slovenia; h Multiphase Chemistry Department, Max Planck Institute for Chemistry, Hahn-Meitner-Weg 1, 55128 Mainz, Germany

**Keywords:** Sarajevo, BiH, aerosol, ETAAS, ICP-MS, BenMAP, ExternE, SAFICA

## Abstract

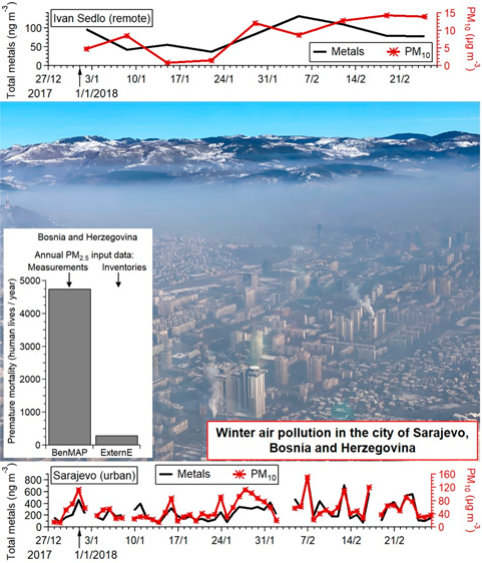

The Sarajevo Canton Winter Field Campaign 2018 (SAFICA) was a project that took place in winter 2017–2018 with an aim to characterize the chemical composition of aerosol in the Sarajevo Canton, Bosnia and Herzegovina (BiH), which has one of the worst air qualities in Europe. This paper presents the first characterization of the metals in PM_10_ (particulate matter aerodynamic diameters ≤10 μm) from continuous filter samples collected during an extended two-months winter period at the urban background Sarajevo and remote Ivan Sedlo sites. We report the results of 18 metals detected by inductively coupled plasma mass spectrometry (ICP-MS) and electrothermal atomic absorption spectrometry (ETAAS). The average mass concentrations of metals were higher at the Sarajevo site than at Ivan Sedlo and ranged from 0.050 ng/m^3^ (Co) to 188 ng/m^3^ (Fe) and from 0.021 ng/m^3^ (Co) to 61.8 ng/m^3^ (Fe), respectively. The BenMAP-CE model was used for estimating the annual BiH health (50% decrease in PM_2.5_ would save 4760+ lives) and economic benefits (costs of $2.29B) of improving the air quality. Additionally, the integrated energy and health assessment with the ExternE model provided an initial estimate of the additional health cost of BiH’s energy system.

## Abstract 2

Air pollution in a global winter hotspot of the Sarajevo Canton, Bosnia and Herzegovina, comes from diverse sources and burdens society’s human health and economy.

## 1 Introduction

Atmospheric particulate matter (PM or aerosol) with aerodynamic diameters ≤ 10 μm (PM_10_) can contain a wide range of chemical components that depend on the emission sources and atmospheric processing.^1−3^ Metals, inorganic ions, organic aerosols, and elemental carbon can be constituents of PM.^4−6^ Even though metals often make up only a relatively small part of aerosol mass,^3^ they are very good tracers of a high number of different sources and processes. Metals originate from mixtures of gas- and particle-bound species from high temperature incineration and combustion of fossil fuels, wood, combustion plants, motor vehicle emissions, as well as from abrasion of tires or brake linings, road dust, crustal weathering, and natural processes.^4,7^ Depending on their size, particles can be deposited in various places of the respiratory tract after inhalation.^8,9^ The inhalation of aerosol metals such as As, Cd, Ni, and Pb can cause damage to the pulmonary, cardiovascular and nervous systems, and liver and enhance mortality.^7,10−12^

The city of Sarajevo is the capital of Bosnia and Herzegovina (BiH) located on the Balkan Peninsula in southeastern Europe with the population of ∼420,000 inhabitants. Sarajevo is situated in the Miljacka river valley at 518 m a.s.l. and surrounded by five major mountains of the Dinaric Alps. Because of Sarajevo’s geographical location and terrain configuration, during winter its tropospheric mixing is frequently low with regularly occurring temperature inversions.^13^ Extensive use of solid fuels during the cold, heating season and an old vehicle fleet coupled with Sarajevo’s orography and meteorology causes the accumulation of pollutants within the city’s plane, leading to episodes of high air pollution.^14^

And yet, despite numerous worldwide studies on atmospheric concentrations and sources of PM constituents, similar studies are scarce in BiH, mostly presenting ambient concentrations of PM_10_, O_3_, NO_2_, NO, and metals in street dust. Available publications discuss air pollution in Sarajevo,^1,14−22^ Tuzla,^23^ Mostar,^24,25^ and Banja Luka.^26^ Delibašić et al.^27^ presented results for metals in street dust of the Federation of BiH, while Ramić et al.^28^ reported data of air pollution biomonitoring in BiH using *Hypogymnia physodes*. Šehbajraktarević et al.^17^ is the only study conducted at the same measuring sites as the study at hand, but with a different focus—heavy metals in atmospheric precipitation. Sulejmanović et al.,^20^ Žero et al.,^1^ and Huremović et al.^21^ focused on short-time measurements of PM metals in the city of Sarajevo, with results showing that metals concentrations are higher during the cold, winter period and household heating season.

Throughout the region of the Western Balkans, it is well established that the energy sector, residential heating, and vehicle fleet contribute to some of the world’s highest levels of winter air pollution.^29^ Recent reports recognized urban atmospheres in the Western Balkans countries such as BiH (Tuzla, Zenica, and Sarajevo), Northern Macedonia (Tetovo and Skopje), and Montenegro (Pljevlja) as some of the most polluted in Europe.^29,30^ Consequently, air pollution was identified as the leading environmental factor driving mortality and disability.^29,31^ Besides gas, the main energy sources in BiH are wood and lignite coal, and their combustion increases the levels of aerosol metals. Individual households in Sarajevo not connected to the city’s central heating system typically heat their homes with wood and other combustible materials (e.g., oil and waste products), which are considered polluting fuels as per Sustainable Development Goals indicator 7.1.2.^32^ Previous research in Kosovo demonstrated how metals found in coal exacerbate public health impacts.^33^ In the case of Sarajevo, both the toxicity and the chemical speciation of PM are unknown. However, from a mass balance perspective and based on the types of public and individual energy sources in Sarajevo, we know that metals aerosolize and become present in air pollution. Primarily, it is important to reduce PM levels and consequently the metals’ levels as they are particle-bound. Although routine measurements are mostly focused on the determinations of metals in PM_10_, it is well-known that these metals are mostly present in the PM_2.5_ fraction;^7^ therefore, in the future planning it would be ideal to focus on PM_2.5_ reduction. For these reasons, it is important to estimate BiH health and economy burdens associated with both observed PM concentrations and the energy sector.

This paper presents the concentrations of ambient PM_10_, their metal content, and possible origin in the Sarajevo Canton during the winter of 2017–2018. This is the first study to report metal content in continuous PM_10_ samples during an extended winter period (two months) with historically the poorest urban air quality from daily samples for Sarajevo and weekly samples for the remote Ivan Sedlo site. Atmospheric mass concentrations of 18 metals are also discussed with factor analysis and meteorological data for an insight into their correlations and emission sources. Finally, this study estimates the influence of only PM_2.5_ to human health in BiH in two ways (from available measurements and emission inventories), to find a lower limit for adverse health effects of air pollution in BiH. The BenMAP-CE model was used with available PM_2.5_ measurements as input for an initial cost-benefit analysis (CBA) of improved air quality, while the ExternE model was used with emission inventories as input to estimate the decrease in mortality and morbidity for energy alternatives.

## 2 Materials and Methods

### 2.1 Sampling Sites

PM_10_ filter samples were collected at two sites in the Sarajevo Canton: Bjelave (urban background site) in the city of Sarajevo and Ivan Sedlo (remote site), both operated by the Federal Hydrometeorological Institute of BiH (FHMIBiH, Supporting Information (SI), Figure S1). The Bjelave site is the headquarters of the FHMIBiH located in the Center Sarajevo municipality^34^ above the river Miljacka valley (altitude: 635 m a.s.l.; 43°52′03′′ N, 18°25′23′′ E). The area is densely populated (population, 53 368) and characterized by single story buildings (family homes) where central heating, wood, coal, and petroleum products are used for domestic heating. The high-volume air sampler (DH77, Digitel-AG, Volketswil, Switzerland) was located in the yard 15 m from FHMIBiH’s main building and 30 m from the nearest two-lane road. A total of 57 daily (24 h), continuous PM_10_ samples were collected on quartz filters (ϕ150 mm, Whatman, QM-A Quartz Microfibre Filters) from December 27, 2017 to February 27, 2018 at an average flow rate of 765 m^3^/day. The average daily temperature at the Sarajevo site during the sampling period was 2.7 °C (minimum: −8.8 °C; maximum: 11.6 °C).

The Ivan Sedlo remote site sits on a mountain ridge ∼45 km southwest from Sarajevo (altitude: 969 m a.s.l.; 43°45′04′′ N, 18°02′10′′ E) far from local anthropogenic emissions. A high-volume air sampler (DH80, Digitel-AG, Volketswil, Switzerland) was located 10 m from the meteorological station Ivan Sedlo operated by the FHMIBiH. A total of nine PM_10_ samples were collected, each for ∼7 days (min 5, max 7 days) using the same filter type as at Bjelave from December 29, 2017 to February 27, 2018 at an average flow rate of 401 m^3^/day. In this paper, the urban background Bjelave and remote Ivan Sedlo sampling sites are referred to as Sarajevo and Ivan Sedlo sites, unless stated otherwise.

### 2.2 Gravimetric and Chemical Analyses of PM_10_ Filter Samples

A detailed description of laboratory analyses is given in the SI section S1. Briefly, the gravimetric measurement of the PM_10_ samples was carried out before and after the field sampling with an analytical microbalance (Mettler Toledo AX205/A) in a balance room with continuously monitored temperature and relative humidity. After gravimetric determination of PM_10_, each filter was cut into several subsamples. One quarter (25%) and one-fifth (20%) of the total filter area were used for the determination of metals by electrothermal atomic absorption spectrometry (ETAAS) and inductively coupled plasma mass spectrometry (ICP-MS), respectively.

ETAAS was used at the University of Sarajevo, BiH, to determine cadmium (Cd), copper (Cu), iron (Fe), vanadium (V), and zinc (Zn) in PM_10_ samples. Filter samples were digested with the mixture of HNO_3_, HF, and H_2_O_2_.^33^ The concentration of metals was determined with an electrothermal atomic absorption spectrometer (model AA240Z, Varian, Mulgrave, Australia) equipped with a graphite furnace (GTA 120) and an autosampler (PSD 120).

ICP-MS was used at the Institute for Medical Research and Occupational Health in Zagreb, Croatia, to determine arsenic (As), barium (Ba), Cd, cerium (Ce), cobalt (Co), cesium (Cs), Cu, Fe, lanthanum (La), manganese (Mn), molybdenum (Mo), nickel (Ni), lead (Pb), rubidium (Rb), strontium (Sr), thallium (Tl), V and Zn in PM_10_ samples. Filter samples were digested with HNO_3_ in a microwave digestion system Ultraclave IV (Milestone Srl, Italy) using an application note for paper filter digestion. Selected isotopes were analyzed with an inductively coupled plasma mass spectrometer (ICP-MS 7500cx Agilent Technologies, Waldbronn, Germany) in collision mode with helium gas for removing the interferences.

Results of metals (Cd, Cu, Fe, V and Zn) measured by both ETAAS and ICP-MS agreed very well for the Sarajevo site samples. In this work, we give an overview of results from both analytical techniques (Table 1 above) and then use ICP-MS results for statistical and other analyses due to the high sensitivity (i.e., lower limits of detection (LODs)) of the ICP-MS, which was particularly important for very low concentrations of metals at the remote Ivan Sedlo site.

**Table 1:** Mass Concentrations of PM_10_ (μg/m^3^) and Metals Measured with ICP-MS and ETAAS (ng/m^3^) from Filter-Collected Samples at Sarajevo and Ivan Sedlo Sites^a^

	Sarajevo				Ivan Sedlo	
	average ± SD	min–max	average ± SD	min–max	average ± SD	min–max
	ICP–MS	ICP–MS	ETAAS	ETAAS	ICP–MS	ICP–MS
PM_10_	51.2 ± 31.3	7.77–151	51.2 ± 31.3	7.70–151	8.44 ± 4.98	0.79–14.34
Element						
As	1.98 ± 1.39	0.09–5.71	n.a.	n.a.	0.36 ± 0.23	0.08–0.81
Ba	5.05 ± 5.95	0.75–46.37	n.a.	n.a.	0.54 ± 0.16	<LOD – 0.88
Cd	0.34 ± 0.23	0.04–0.98	0.58 ± 0.38	0.07–1.59	0.077 ± 0.045	0.020–0.142
Ce	0.17 ± 0.14	0.03–0.84	n.a.	n.a.	0.046 ± 0.059	<LOD – 0.180
Co	0.050 ± 0.031	0.010–0.158	n.a.	n.a.	0.022 ± 0.011	0.010–0.045
Cs	0.097 ± 0.075	0.011–0.345	n.a.	n.a.	0.036 ± 0.023	0.010–0.084
Cu	4.41 ± 3.16	0.67–15.69	4.45 ± 4.58	<LOD – 19.70	0.63 ± 0.21	0.33–0.90
Fe	188 ± 127	36.0–674	190 ± 156	<LOD – 815	63.0 ± 28.1	27.8–115.3
La	0.081 ± 0.070	0.015–0.405	n.a.	n.a.	0.025 ± 0.030	<LOD – 0.090
Mn	6.04 ± 4.28	1.03–20.01	n.a.	n.a.	2.71 ± 1.19	1.17–4.67
Mo	0.20 ± 0.13	<LOD – 0.62	n.a.	n.a.	0.034 ± 0.017	<LOD – 0.060
Ni	0.69 ± 0.56	0.11–3.64	n.a.	n.a.	0.26 ± 0.10	0.14–0.44
Pb	8.01 ± 5.69	0.97–24.63	n.a.	n.a.	2.69 ± 1.84	0.53–5.78
Rb	2.19 ± 1.49	0.24–6.54	n.a.	n.a.	0.36 ± 0.10	0.25–0.50
Sr	0.96 ± 1.91	<LOD – 14.99	n.a.	n.a.	0.26 ± 0.27	<LOD – 0.92
Tl	0.083 ± 0.091	0.006–0.517	n.a.	n.a.	0.046 ± 0.035	0.007–0.113
V	0.94 ± 1.09	0.09–7.27	1.08 ± 1.41	<LOD – 9.16	0.29 ± 0.20	0.08–0.81
Zn	35.1 ± 23.8	5.50–106	45.6 ± 64.8	<LOD – 389	8.13 ± 3.69	3.26–13.51

a Notation: n.a., not available; LOD, limit of detection.

### 2.3 US EPA BenMAP Cost-Benefit Analysis

The US Environmental Protection Agency (US EPA) Environmental Benefits Mapping and Analysis Program—Community Edition (BenMAP–CE) software estimates the health effects of air pollution by using ambient concentrations of PM_2.5_ and ozone as inputs. The BenMAP-CE tool was used to conduct cost-benefit analysis (CBA) for BiH for the scenario of improved air quality with the reduction of PM_2.5_. A detailed description of the BenMAP-CE parameters is given in the SI section S2. Briefly, we used an estimated value of statistical life and the function of affecting health. The function of health impact was calculated based on the real, available PM_2.5_ measurements in three major BiH cities (Sarajevo, Tuzla, and Zenica) during 2016 (SI Figures S2 and S3) to give an estimate of annual mortality in BiH due to the air pollution (SI Figure S4). Also calculated was the alternative scenario of PM_2.5_ reduction in the 5–95% range, as well as the resulting decrease in annual BiH mortality.

In this study, we used only PM_2.5_ as BenMAP input to estimate the effect of PM only and all input BenMAP data was taken from available FHMIBiH measurements.^35^ Ozone relevance in the summer^36,37^ is not within the scope of the winter measurements reported in this paper. Lack of ozone is not expected to influence BenMAP results as the poorest BiH air quality is during the cold season not characterized by elevated ozone.^36,37^ Similarly, this study reports the results of metals for PM_10_ filter samples, and the vast majority of PM online measurements conducted by FHMIBiH as a part of BiH air quality network are PM_10_ ones. PM_2.5_ online measurements are conducted only in the city of Tuzla. Therefore, in this study, available PM_2.5_ online measurements were used for Tuzla. For Sarajevo and Zenica the following approximation was made: PM_10, online_ = PM_2.5, BenMAP input_ (i.e., PM_2.5, BenMAP input_/PM_10, online_ = 1). Again, this is a reasonable assumption for BiH high winter air pollution that is due to incomplete combustion emissions known to produce ultrafine and fine PM (PM_0.1_ and PM_2.5_, respectively),^36,37^ as recently corroborated by number concentration and size distribution measurements in Sarajevo.^38^

### 2.4 Energy Alternatives

To estimate premature reduction in life expectancy and respiratory illnesses, we use a top-down approach implementing an occupational and air-pollution-related risk model Externalities of Energy (ExternE). ExternE, an energy and health modeling tool,^39^ was applied to evaluate the health impacts of energy usage in BiH, using a similar approach as described in Kittner et al. (2018).^33^ This approach also accounts for premature deaths and major and minor respiratory illnesses based on the energy composition. The approach considers the primary energy mix of BiH and provides an initial estimation of the difference between the “business-as-usual current energy mix”, and “potential alternative energy sources” scenarios. A detailed description of the ExternE modeling tool is given in the SI section S3. Briefly, the model estimates health impacts attributable to air pollution for each energy technology scenario as reported by the International Energy Agency expressed per kWh or unit energy in MJ. Reported mortality is based on emission factors for PM_2.5_, sulfur dioxide, nitrogen oxides, and ozone, and does not necessarily include the metals that are present.^39,40^

## 3 Results and Discussion

### 3.1 Ambient concentrations of PM_10_ and its metal content

The summary statistics of metals’ mass concentrations in PM_10_ filter samples collected at Sarajevo and Ivan Sedlo sites are presented in Table 1. The daily average PM_10_ mass concentration during the sampling period in Sarajevo ranged from 8 to 151 μg/m^3^ with an average of 51 μg/m^3^. For the period reported here, the PM_10_ daily limit value of 50 μg/m^3^ set by European^41^ and BiH legislation^42^ was exceeded on 27 days (6 days >100 μg/m^3^). A recent study by Huremović et al.^21^ compared daily average PM_10_ mass concentrations reported also in this study with those measured during the heating seasons from 2010 to 2019 at a different urban site in Sarajevo (Pofalići) and found days with extraordinary high values that sometimes exceeded 400 μg/m^3^. The average daily PM_10_ mass concentration at Ivan Sedlo site was 8 μg/m^3^, ∼6× lower than in Sarajevo.

The European Environment Agency reported a significant PM_10_ decrease in Europe during the period from 2000 to 2018 as well as a reduction in the concentrations of As, Cd, Ni, and Pb for the same period.^43^ However, the same trends were not found for PM_10_ and elements measured during winter seasons 2010–2019 in Sarajevo.^21^ PM_10_ results similar to the Sarajevo ones in this study were reported in Turkey (Tuzla region in the vicinity of Istanbul),^44^ in the industrial city of Elefsis in Greece,^45^ in urban areas of southeast Italy^46^ during 2003–2010, and in Belgrade, Serbia.^47^ For Belgrade, Joksić et al.^47^ reported average daily PM_10_ of 37 μg/m^3^ and 44 μg/m^3^ during spring and summer of 2007, respectively, Mijić et al.^48^ values of 70 μg/m^3^ for urban Belgrade area, and Perišić et al.^49^ the range of values of 32–81 μg/m^3^ for the wider Belgrade area. The maximum PM_10_ value of 151 μg/m^3^ at the Sarajevo site in this study was similar to those obtained for Ahvaz, the capital of the Khuzestan Province in Iran during winter 2013 (average 189 μg/m^3^).^50^ However, the Ahvaz, Iran values were for PM_10_ measurements that included periods during dust storms,^50^ and there is no evidence of dust in the BiH samples during the winter campaign in this study. The Italian study^46^ also found >3× higher daily average PM_10_ values at remote Italian locations, compared to the remote Ivan Sedlo site.

**Figure 1:**
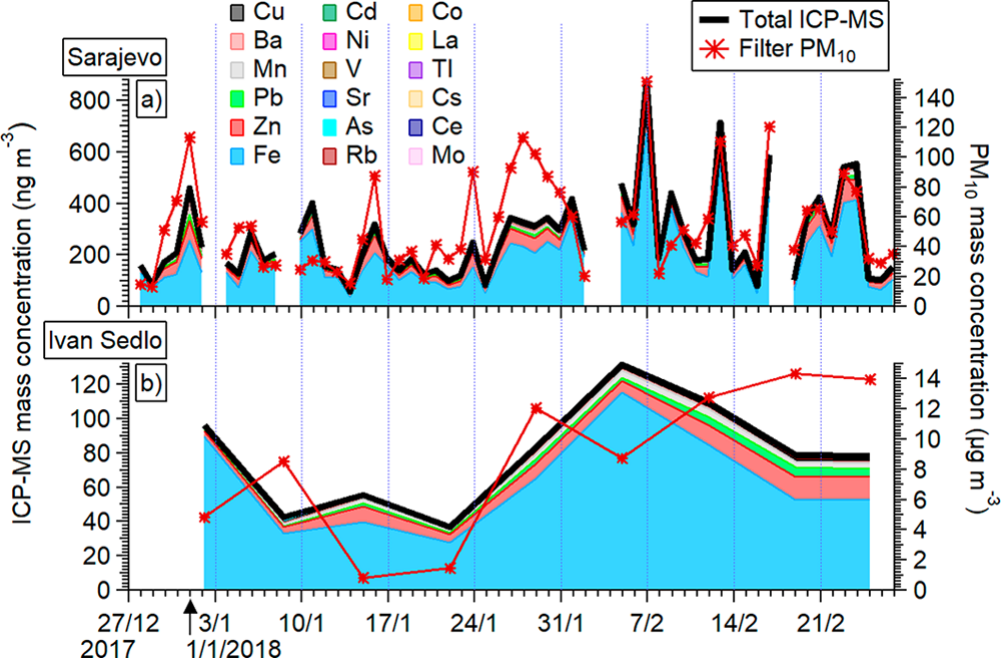
Time series of individual metals and their total measured by the ICP-MS analytical technique in PM_10_ (also shown) filter samples collected at the Sarajevo urban background site (a) and the Ivan Sedlo remote site (b).

Figure 1 shows the time series of PM_10_ and ICP-MS metals at two sites. Levels of metals were significantly higher in Sarajevo compared to those in Ivan Sedlo (Table 1 and Figure 1), as expected: for most metals, their ambient concentrations were 2–3× higher; and for Zn, Cd, and Sr, 4×; As, 5×; Rb and Mo, 6×; Cu, 7×; and for Ba, 10× higher in Sarajevo. Previously, authors reported metals concentrations in atmospheric precipitation at the same two sites^17^ and found higher Ni and Mn concentrations in Sarajevo due to urban vehicular emissions that are absent at Ivan Sedlo.

**Figure 2:**
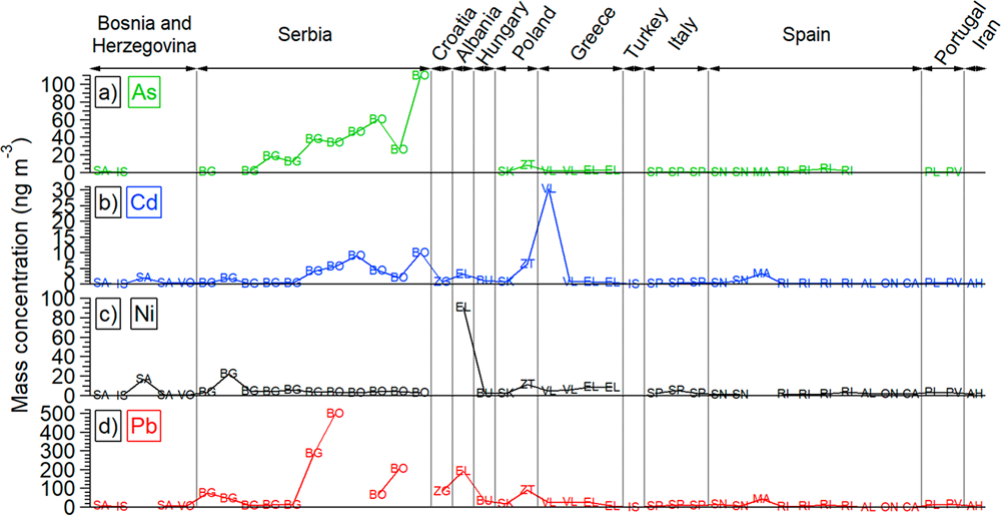
Comparison of PM_10_ ambient loadings in this study with other locations for As, Cd, Ni, and Pb as metals regulated by EU legislation. Each site is marked by its abbreviation and the first two points marked SA and IS are for Sarajevo and Ivan Sedlo, respectively, sites from this study. Detailed description of all measurement sites and measured metals’ values is given in Tables S2–S3.

The chemical composition of this study is compared to the values reported for sites in other cities in which the same analytical techniques were used (Figure 2 and Tables S2–S3). Obtained concentrations of As are comparable to results for sampling sites in Poland,^51^ Greece,^52^ Spain,^53^ and Serbia.^49^ The concentrations of Cd and Pb are lower than these reported for Poland,^51,54^ Hungary,^55^ Greece,^52^ Spain,^56^ Italy,^46^ Serbia,^47−49,57^ and Croatia.^58^ The concentration for Ni reported for Poland,^51,54^ Hungary,^55^ Portugal,^59^ Greece,^52^ Spain,^56,60^ Italy,^46,53^ Serbia,^47,48,57^ Albania,^61^ and Iran^50^ are higher than the values obtained for the Sarajevo and Ivan Sedlo sampling sites in this study.

Figure S5 shows average mass concentrations of metals with their contributions to the total ICP-MS metals. The highest and lowest amounts at both sites were found for Fe and Co, respectively. Mineral dust is usually the major source of Fe, however in this study the likely sources are nonexhaust traffic emissions (brake and body wear) and road dust resuspension.^62^ The results for Co obtained in this study are similar to the results for Co obtained in the study by Fomba et al.^7^ The metals’ decreasing order of concentrations in the total ICP-MS mass for the Sarajevo site was Fe > Zn > Pb > Mn > Ba > Cu > Rb = As > V = Sr > Ni > Cd > Mo > Ce > Tl > Cs > La > Co. Similar results were found for Ivan Sedlo, where the metals’ decreasing order of concentration was Fe > Zn > Mn > Pb > Cu > Ba > Rb = As > V > Ni > Sr > Cd > Ce = Tl > Cs > Mo > La > Co (Figure S5).

Although the sum of the investigated metals did not exceed 1% of total PM_10_ mass (1% of PM mass at Ivan Sedlo and 0.5% at Sarajevo site), some are important for public health due to their adverse health impacts. For example, Pb, Ni, Cd, and As can have a harmful impact on humans, and therefore the European legislation has set their annual PM_10_ limit (Pb) and target (Ni, Cd, and As) values at 500, 20, 5, and 6 ng/m^3^, respectively. The concentrations obtained in this study for Pb, Ni, Cd, and As were below the values of EU regulations,^63−65^ which is consistent with the fact that the As, Cd, and Pb concentrations are generally low in Europe.^66^

### 3.2 Factor Analysis of PM_10_ Metal Content

PM metals can be used as indicators of their emission sources, that is, origin.^67^ Fossil fuel combustion is one of the main anthropogenic aerosol sources. Particle-bound As, Cd, Pb, Co, Cu, and Mo are emitted by coal combustion, while V, Ni, Pb are emitted mostly by heavy oil combustion sources.^68,69^ Cu, Zn, and Pb are often associated with traffic emissions and road dust,^7,70^ and as reported by Gugamsetty et al.,^70^ Zn and Cu are often used as tracers for tire and break wear, respectively. Higher concentrations of Cu at Sarajevo site could be from the wear of vehicles’ brakes.^49^ Cd occurs at high temperatures during the combustion of coal and oil.^70^ All of these emission sources could be responsible for metals’ values in Sarajevo.

The correlation analysis revealed very strong correlations between most metals measured at the Sarajevo site (Table S4), while at the Ivan Sedlo site the strongest correlations existed between several metals, grouped as V–Ni–Sr, Cu–Zn–As–Rb–Cd–Cs–Tl–Pb, and La–Ce (Table S5). Huremović et al.^21^ found a very strong correlation (*r* > 0.824) between Cd and Pb in PM_10_ at the urban Pofalići site in Sarajevo, suggesting that Cd and Pb originated from the same pollution source(s), such as waste incineration, burning of treated and/or painted wood, and fossil fuel combustion.^71,72^

Factor analysis of the metals’ results at the Sarajevo site was used to extract factors that could provide additional insight into the sources of particulate metals. Note that the factor analysis of Sarajevo site metals is not a full source apportionment of PM_10_ and refers only to the measured metals since they are an extremely small part of PM_10_ (∼1%). Nevertheless, this is the first step to get an idea of the processes that affect the measured metals in the city of Sarajevo, as in similar factor analyses^73,74^ or source apportionment^67,75−77^ studies that look only at particulate metals’ content. Factor loadings were calculated with the STATISTICA program package (ver. 13.0., Dell Inc.) using principal component extraction, and eigenvalues were rotated by the normalized varimax method. Factor loadings greater than 0.6 were considered significant (Table 2).

**Table 2:** Factor Loadings of Elements Mass Concentrations at the Sarajevo Site (Extraction: Principal Components, Loadings > 0.6 Are Marked Bold)

	factor 1	factor 2	factor 3	factor 4
V	0.181	0.329	0.004	**0.832**
Mn	**0.840**	0.359	0.083	0.289
Fe	0.577	**0.648**	0.087	0.433
Co	0.551	**0.672**	0.114	0.416
Ni	0.277	0.435	0.112	**0.795**
Cu	0.423	0.270	0.569	0.578
Zn	**0.799**	0.050	0.260	0.493
As	**0.706**	–0.044	0.278	0.572
Rb	0.525	–0.023	0.512	0.561
Sr	0.045	0.116	**0.956**	0.024
Mo	0.349	0.187	0.236	**0.730**
Cd	**0.713**	0.002	0.339	0.543
Cs	**0.930**	0.082	0.139	0.281
Ba	0.166	0.177	**0.927**	0.218
La	–0.048	**0.949**	0.105	0.161
Ce	0.001	**0.943**	0.159	0.116
Tl	**0.939**	0.026	–0.056	–0.009
Pb	**0.714**	0.050	0.368	0.531
eigenvalue	10.89	2.58	1.89	1.02
% Total variance	60.5	14.4	10.5	5.7

**Figure 3:**
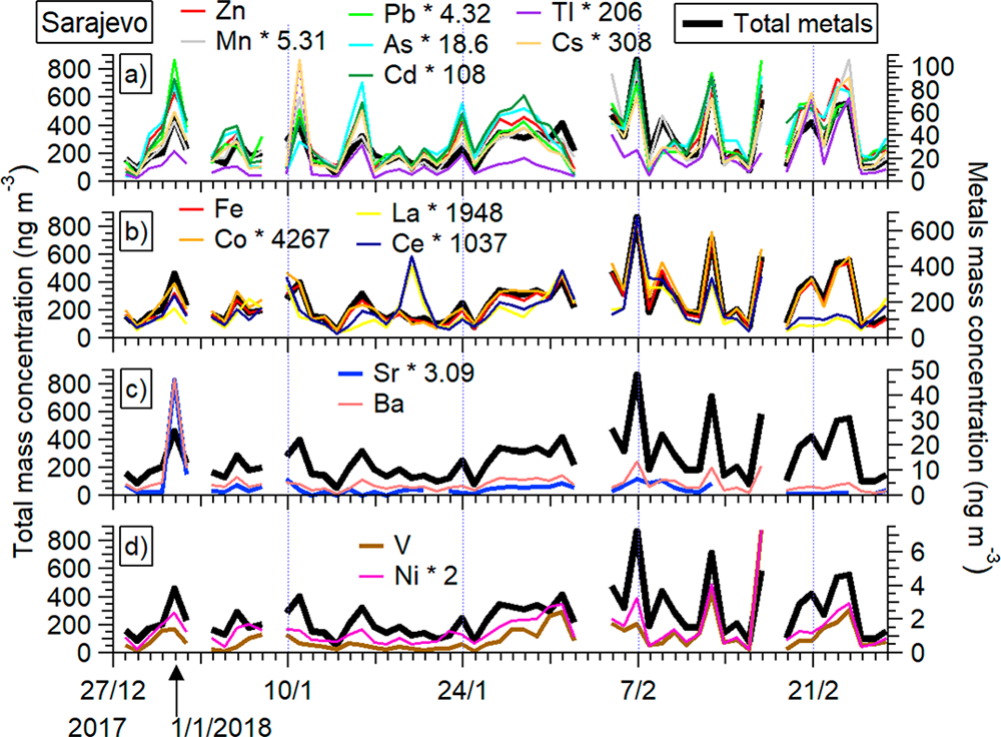
Sarajevo site mass concentrations of metals grouped as the results of factor analysis (i.e., PM_10_ metals in each factor): (a) factor 1 (Zn, Mn, Pb, As, Cd, Tl and Cs); (b) factor 2 (Fe, Co, La and Ce); (c) factor 3 (Sr and Ba); and (d) factor 4 (V and Ni). Metals in each factor are scaled to the metal of highest abundance for clarity of presentation (scaling factors are also shown). Also shown is total ICP-MS metals mass concentration.

Factor analysis resulted in four factors with eigenvalues higher than 1, and the time series of groups of metals in each factor is shown in Figure 3. Factor 1 includes Mn, Zn, As, Cd, Cs, Tl, and Pb with total variance explained by this factor of 60.5% (Figure 3a). As, Cd, and Pb are known as toxic and carcinogenic elements which have an anthropogenic origin from the burning of treated wood and waste, as well as industrial emissions, and Zn and Mn can also have the same sources. Tl is related to a specific source such as cement production. Cement production takes place at several locations in BiH and the closest cement factory is about 50 km away from Sarajevo. Thus, some elements may have been transported to the Sarajevo atmosphere from the wider area by resuspension of PM. Therefore, factor 1 represents a mixed anthropogenic source that is mainly related to domestic heating and also to traffic and industry. Žero et al. (2017)^1^ came to very similar results by analyzing PM_10_ from urban and rural sites in the Sarajevo Canton, BiH. The enrichment factor analysis performed by the authors showed that potential sources of PM are domestic heating, oil burning, and agricultural activities. Factor 2 includes Fe, Co, La, and Ce, which are crustal elements and stem from natural sources (Figure 3b) as confirmed with enrichment factor analysis (SI section S4 and Figure S6). Ba and Sr are in factor 3, and we note that samples with high content of Ba also have a high content of Sr (Figure 3c). The highest values of Ba and Sr are found for December 31, 2017 to January 1, 2018 and can be explained with elevated PM_10_ values due to New Year 2018 celebrations, more precisely fireworks. Similar results were found in a recent US study^78^ of PM_2.5_ components around July 4, which identified a significant increase of Ba and Sr in PM_2.5_ on July 4, because Cu, Ba, and Sr are used extensively in pyrotechnic coloring and glitter effects. Factor 4 included V and Ni, which are emitted from heavy oil combustion (Figure 3d). The presence of V and Ni points to the widespread use of heavy oils as energy sources in the city of Sarajevo, possibly in transport and heating, as there is no industry in the city of Sarajevo that could explain elevated levels of particulate V and Ni. Recent research in Sarajevo identified similar sources of particulate polycyclic aromatic hydrocarbons (PAHs), that is, combustion of gasoline and diesel from traffic, as well as burning of heavy oil, wood, and coal.^22^ We did not perform factor analysis for Ivan Sedlo site metals’ results due to the small number of collected samples (9). Nevertheless, the temporal variations of PM_10_ metals that were grouped together in four factors at the Sarajevo site, do show a similar behavior also at the Ivan Sedlo site (Figure S7).

### 3.3 The Influences of Meteorological Conditions on PM_10_ Metal Content

**Figure 4:**
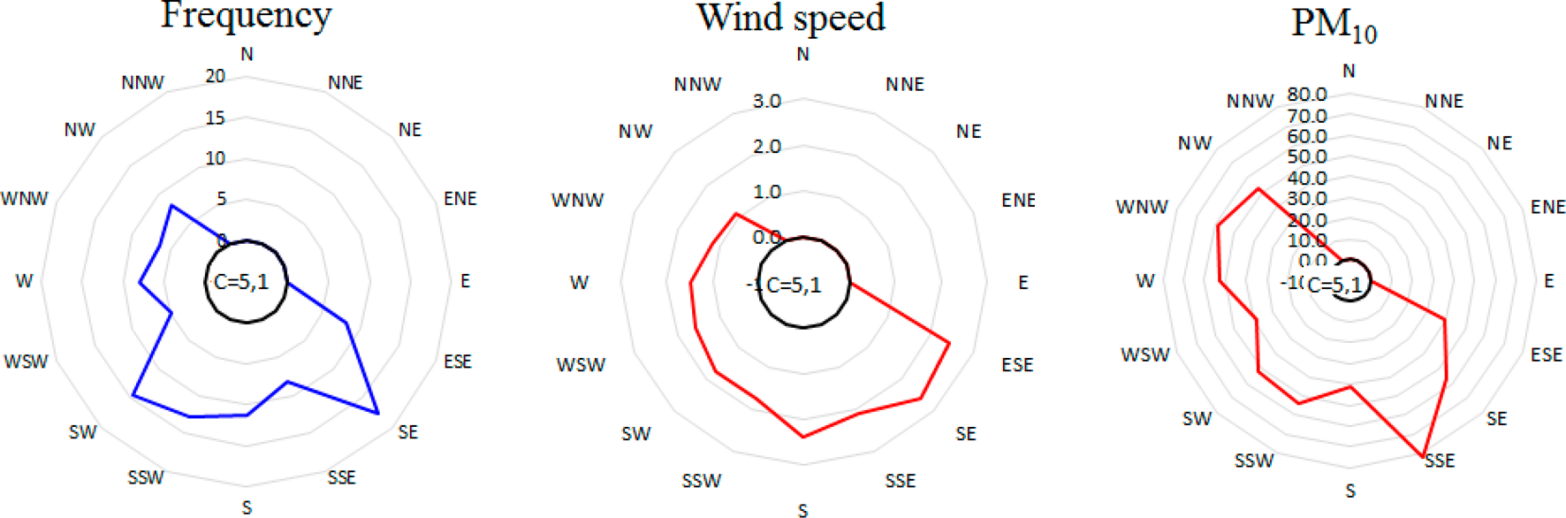
Distribution of wind frequency, wind speed, and PM_10_ mass concentrations according to the wind directions for the Sarajevo sampling site.

To evaluate the major directions of air pollution origin for the Sarajevo sampling site, wind patterns are correlated with the concentrations of measured metals. Wind frequency, wind speed, and distribution of PM_10_ mass concentrations are presented in Figure 4. The meteorological parameters are routinely measured at the Bjelave site by FHMIBiH.^79,80^

The major loadings for PM_10_ come from the southeast, south and west, the direction where the majority of urban Sarajevo is located, while none of it came from north and northeast. Distributions of individual metals in PM_10_ according to the direction of air paths for the sampling site Sarajevo are presented in Figures S8–S11. The results show that mass concentrations of metals have a different distribution than PM_10_ and some metals are grouped together with similar patterns. The city landfill as one of the potential sources is located on the western part of the city. The highest concentrations of Pb, Cd, As, Zn, Mn, Tl, and Cs, metals also grouped as factor 1, were found with air masses flowing from the west-northwest direction (Figure S8). The elevated values for these metals from the south direction came from the city center, confirming traffic and household heating (e.g., burning of wood and waste) sources. Fe, Co, La, and Ce that were grouped as factor 2 also show a distinct contribution to the air masses flowing from the southwest direction (Figure S9). The completely different distribution of Ba and Sr with highest values arriving from the south–southeast direction implicates a separate source, as confirmed by separate factor 3 in factor analysis (Figure S10). V and Ni, grouped as factor 4, also have a similar distribution with the major path of pollution equally coming from the south and the west (Figure S11). In summary, identified wind patterns for metals’ ambient loadings at the Sarajevo site confirm the conclusions drawn in their factor analysis: metals grouped together in each of four factors also show different meteorological patterns.

### 3.4 Benefit Analysis Model–Bottom Up Approach

Recent research recognized the transition metals (e.g., Cu, Mn, Fe, V, and Ni) as key contributors to the aerosol oxidative potential (OP) and also suggested OP as a better indicator of air pollutions’ adverse health effects than PM_2.5_. Nevertheless, we wanted to look at another limiting case and estimate the influence of total PM_2.5_ to human health in BiH from available measurements and emission inventories (next section), as a lower limit for health effects. The BenMAP tool enabled a successful initial CBA for BiH with the scenario of improved air quality, based on the reduction of PM_2.5_. Note that this is an initial BenMAP analysis for the purpose of this research, and refined analysis should be made once more air quality and other relevant data is available, such as for example, BiH emission inventories and online measurements of PM_2.5_. Sarajevo, Tuzla, and Zenica are cities with increased PM_2.5_, and there is ample evidence of PM_2.5_ correlation with acute and chronic mortality worldwide, starting with the pioneering Harvard Six Cities Study.^81^ The annual average mass concentrations of PM_2.5_ for Sarajevo, Tuzla, and Zenica during 2016 were 58 μg/m^3^, 62 μg/m^3^, and 70 μg/m^3^, respectively (Figure S2). Those values for PM_2.5_ were significantly higher than the EU limit value of 25 μg/m^3^. All three cities experienced increased daily average values of PM_2.5_ during winter, often at hazardous levels (>250 μg/m^3^), while for the rest of the year PM_2.5_ was at the limit value. BenMAP CBA was purposely conducted for the year 2016, as opposed to years 2017 or 2018 that would include the SAFICA campaign winter of 2017–2018. Namely, the SAFICA winter of 2017–2018 was characterized with unusually low ambient loadings of PM in the city of Sarajevo. Average daily PM_10_ measured online at the Bjelave site during the winter of 2016–2017 in the months of December through February was 116.6 μg/m^3^ (minimum, 6.9 μg/m^3^; maximum, 622.8 μg/m^3^), while the same value for SAFICA winter of 2017–2018 was 54.1 μg/m^3^ (minimum, 10.4 μg/m^3^; maximum, 214.3 μg/m^3^).^82−84^ The following winters in Sarajevo had PM concentrations similar to the winter of 2016–2017. Furthermore, BiH wide PM values measured by FHMIBiH during the winter of 2019–2020 were some of the highest ever recorded.^85^ Therefore, to simulate historically typical conditions of BiH air pollution with BenMAP and ExternE tools (detailed in section 3.4 below), we choose the year 2016.

**Figure 5:**
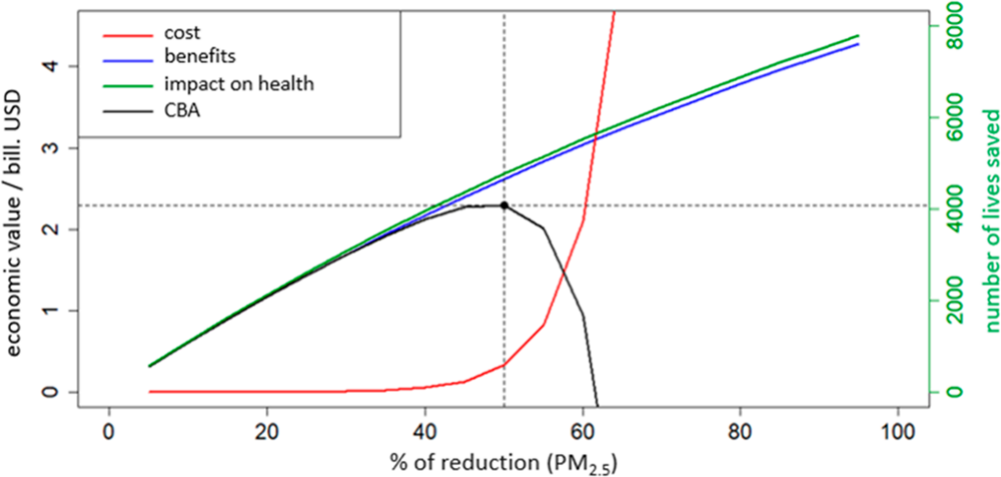
Cost and benefit analysis: Dependence on the PM_2.5_ reduction percentage and the cost to the BiH society (red), benefits (blue), impact on health (green), and benefit and cost differentials (CBA, black). The ideal reduction is defined as the maximum of the CBA curve and is 50% for BiH.

The CBA made using BenMAP-CE points out that implementation of new air quality improvement strategies^86^ would bring significant health and economic benefits to the BiH. BenMAP-CE CBA showed that it would be ideal to decrease PM_2.5_ by 50%, to an annual average of 17.5 μg/m^3^. The mentioned 50% decrease in PM_2.5_ would annually save more than 4760 lives in BiH, bringing economic benefits of $2.29B, which is shown in Figure 5.

### 3.5 Energy Alternatives–Top Down Approach

In 2016, BiH accounted for 9.9 × 10^16^ J of primary energy.^87^ Wood and other biomass materials accounted for approximately 5.4 × 10^16^ J of primary energy across the entire country. Summary statistics from the US Energy Information Administration (EIA) report approximately 18 million metric tonnes of CO_2_ with about 7.7 × 10^4^ MJ/person.^87^ Electricity was primarily generated from the fossil fuel sector, accounting for 17 billion kWh in 2016, with 5.5 billion kWh from hydropower plants. Most of the identified health impacts were from transportation, household heating, and other industrial combustion sources, as well as electricity generation by coal power plants.

The additional annual air-pollution-attributable morbidity and mortality based on the energy sector highlights the deaths from air pollution related risk calculated for BiH based on the best available data for coal, natural gas, and biomass consumption. The ExternE energy and health modeling software assumes a population density of 60 people/km^2^ and does not include source-specific metals in the PM burden. The ExternE model results estimate the annual health effects in BiH due to lignite, coal, wood, and other energy source reliance to be 300 (90–1400) premature deaths, 3100 (800–13000) serious respiratory illness cases, and 190000 (47000–750000) minor respiratory illness cases. Additionally, the recent International Energy Agency report on energy consumption data suggests there was a 126% increase in BiH annual national combustion of waste products between 2016 and 2018, indicating that without further mitigation action, these premature deaths and illness estimates could increase in future years.^88^ One limitation is the assumption of a linear relationship between exposure to PM emissions and premature deaths based on energy consumption. However, a linear relationship is likely the best available estimate given that background PM levels are high enough to appear in the linear portion of the concentration–response curve and there is limited empirical data available in the energy sector to link the air pollution measurements directly with the energy sector.

In 2016, BiH utilized 6.3 × 10^10^ MJ of oil products, 4 × 10^7^ MJ of electricity (∼60% from coal), 2 × 10^7^ MJ of biofuels and waste energy, and 1.6 × 10^7^ MJ of industrial coal. This energy highlights the opportunity cost of using oil, biofuels, and waste energy in the estimation of premature deaths from air pollution, as many of these are poorly represented. Yet there are reports of municipal and household burning of trash and biomass in Sarajevo and BiH that is detected in this analysis that indicate the (90–1400) premature deaths/year as estimated by ExternE model may serve as a lower estimated range.

## 4 Implications for Sarajevo, BiH, and the Western Balkans region

This study indicates that the sources of metals in the Sarajevo Canton come from the combustion of a diverse mix of solid (e.g., wood, pellets, and waste) and liquid fuels. The study detected some fuels that are intuitive for Sarajevo, which has numerous households heated by wood products and a centralized heating system powered by gas (a nonpolluting source) October through April. However, we also saw the evidence of energy usage that has no intuitive sources, such as heavy oils. Air pollution sources may be significantly different in other, heavily industrialized BiH urban centers with industry powered by coal such as Tuzla and Zenica, where the use of coal in households could also be more prevalent than in Sarajevo. BenMAP and ExternE approaches used to estimate the annual premature mortality in BiH gave significantly different results of 4760 and 300, respectively, human lives lost due to air pollution. The most likely reason is that BenMAP inputs are PM measurements data, while ExternE only considers formally reported energy consumption data with their assumed emission factors. This implies that informal biomass burning for heating or cooking (and possibly other PM sources) is underreported in BiH and leads to significantly higher PM levels. In summary, there is a dire need for future continuous, extended measurement studies and physicochemical characterization of atmospheric pollution, as only measurements can constrain models and reduce their uncertainties. These findings are important for understanding the current air pollution crisis in BiH urban centers and are also applicable to the Western Balkans region due to the use of similar energy sources and society life style.

Finally, to alleviate negative PM health impacts in BiH, nonpolluting energy options should be planned and implemented. In BiH, there is the potential to utilize low-pollution and clean energy sources such as electricity generated from solar photovoltaics and wind power. Low-carbon electricity could offset nearly 99% of the additional air-pollution attributable morbidity and mortality statistics–particularly in the electricity sector. However, it is crucial to make new energy solutions available to the general population through affordable prices and subsidies (i.e., minimal personal financial investment per individual household). Only in this case will the population of BiH be encouraged to move to more environmentally friendly solutions in terms of heating and energy use in general. In the transportation sector, electric vehicles could be used to avoid the negative impacts of combustion, including PM emission. If the transportation and heating sectors were electrified through the use of electric vehicles and electric heat pumps, there could be further reductions due to avoided use of fossil fuels, wood fuels, and waste in numerous individual households.

## Supporting Information Available

Analyses of PM_10_ filter samples (Text S1): mass (Text S1.1) and chemical analyses (Text S1.2); BenMAP-CE and ExternE parameters (Texts S2–3); recoveries of certified reference materials (Table S1); comparison of PM_10_ metals (Tables S2–S3); Pearson’s correlation matrices at Sarajevo (Table S4) and Ivan Sedlo (Table S5) sites; PM_10_ sampling sites (Figure S1); BenMAP: measured PM_2.5_ (Figure S2); PM_2.5_ heatmap result (Figure S3); and mortality result (Figure S4); metals average concentrations and contributions (Figure S5); enrichment factors (Figure S6); grouped metals at Ivan Sedlo site (Figure S7); and distribution of metals with wind patterns at Sarajevo site (Figures S8–S11) (PDF)
