# Supplementary material for: New Insight into the Measurements of Particle-Bound Metals in the Urban and Remote Atmospheres of the Sarajevo Canton and Modeled Impacts of Particulate Air Pollution in Bosnia and Herzegovina
Source: Environ Sci Technol. 2022 Mar 2;56(11):7052–62. doi: 10.1021/acs.est.1c07037 (PMC9178787; doi:10.1021/acs.est.1c07037)
Supplement: Supplementary file 1 — es1c07037_si_001.pdf [file es1c07037_si_001.pdf]

Supporting Information for:

New insight into the measurements of particle-bound metals in the urban and remote atmospheres of the Sarajevo Canton and modeled impacts of particulate air pollution in Bosnia and Herzegovina

Sabina Žero, Silva Žužul, Jasna Huremović, Gordana Pehnec, Ivan Bešlić, Jasmina Rinkovec, Ranka Godec, Noah Kittner, Karla Pavlović, Nino Požar, Juan J. Castillo, Sergio Sanchez, Manousos I. Manousakas, Markus Furger, Andre S.H. Prevot, Griša Močnik, Katja Džepina*

AUTHOR INFORMATION

Jasna Huremović and Sabina Žero - Department of Chemistry, Faculty of Science, University of Sarajevo, 71000 Sarajevo, Bosnia and Herzegovina

Silva Žužul, Gordana Pehnec, Ivan Bešlić, Jasmina Rinkovec, and Ranka Godec – Environmental Hygiene Unit, Institute for Medical Research and Occupational Health, 10000 Zagreb, Croatia

Noah Kittner - Department of Environmental Sciences and Engineering, University of North Carolina at Chapel Hill, Chapel Hill, NC 27599-7400, USA

Karla Pavlović and Nino Požar - Department of Biotechnology, University of Rijeka, 51000 Rijeka, Croatia

Juan J. Castillo - Clean Air Institute, Washington, DC 20005, USA (Now at Pan American Health Organization, Washington, DC 20037, USA)

Sergio Sanchez - Clean Air Institute, Washington, DC 20005, USA (Now at Environmental Defense Fund, Washington, DC 20009, USA)

Manousos I. Manousakas, Markus Furger and Andre S.H. Prevot - Laboratory of Atmospheric Chemistry, Paul Scherrer Institute, 5232 Villigen PSI, Switzerland

Griša Močnik - Center for Atmospheric Research, University of Nova Gorica, SI-5270 Ajdovščina, Slovenia

Katja Džepina - Center for Atmospheric Research, University of Nova Gorica, SI-5270 Ajdovščina, Slovenia; Multiphase Chemistry Department, Max Planck Institute for Chemistry, Hahn-Meitner-Weg 1, 55128 Mainz, Germany; Laboratory of Atmospheric Chemistry, Paul Scherrer Institute, 5232 Villigen PSI, Switzerland

***Corresponding author:** Katja Džepina <katja.dzepina@ung.si>

Supporting Information summary:

Total number of SI pages: 25

Number of pages of SI text: 5

Number of SI tables: 5

Number of SI figures: 11

List of Supporting Information tables and figures:

Table S1. Recoveries of certified reference materials used in this study.

Table S2. Overview of sampling sites, periods and analytical techniques used in metals' measurements in this study and other locations presented in Table S3.

Table S3. Comparison of PM_{10} metals mass concentrations in this study with other locations.

Table S4. Pearson's correlation matrix between the metal concentrations in PM_{10} samples collected at Sarajevo (N=57). Statistically significant coefficients ($p < 0.001$) are marked red.

Table S5. Pearson's correlation matrix between the metal concentrations in PM_{10} samples collected at Ivan Sedlo (N=9). Statistically significant coefficients ($p < 0.001$) are marked red.

Figure S1. The position of two PM_{10} sampling sites, urban background Bjelave site within the city of Sarajevo and remote Ivan Sedlo site at the mountain ridge 45 km away within the regional (left panel) and local geography (right panel). Created in the US Geological Survey Topographic Maps (https://ngmdb.usgs.gov/topoview/).

Figure S2. Measured daily average PM mass concentrations during 2016 for (a) Sarajevo (PM_{10}); (b) Tuzla ($PM_{2.5}$); and (c) Zenica (PM_{10}). Lines indicate $PM_{2.5}$ daily recommended value (25 $\mu g/m^3$) and the one for hazardous level (250 µg/m3) (EU Directive 2008/50/EC). Names of different measurement sites in each of three cities are noted in each panel. For each town 4 sampling sites were defined, shown in various colors in panels. All data were taken from the Federal Hydrometeorological Institute of Bosnia and Herzegovina, available online[33].

Figure S3. Heatmap of average annual $PM_{2.5}$ mass concentrations in BiH during 2016. Heatmap $PM_{2.5}$ results were produced by BenMAP tool with $PM_{2.5}$ BiH measurements (also shown in Figure S2) as input data.

Figure S4. BenMAP results of mortality in BiH from: (a) lung cancer; (b) cerebrovascular diseases; (c) ischemic heart disease; and (d) chronic obstructive pulmonary disease.

Figure S5. Average mass concentrations of all metals measured by ICP-MS analytical technique at the Sarajevo urban background site and the Ivan Sedlo remote site. Also shown are the percent contributions of each metal to the total ICP-MS metal mass.

Figure S6. Crustal enrichment factors of elements analyzed from PM_{10} samples collected at Sarajevo site (EF=1 is marked as red line).

Figure S7. Ivan Sedlo site mass concentrations of metals grouped as four factors results of factor analysis for Sarajevo site (PM_{10} metals in each factor): a) Factor 1 (Zn, Mn, Pb, As, Cd, Tl and Cs); b) Factor 2 (Fe, Co, La and Ce); c) Factor 3 (Sr and Ba); and d) Factor 4 (V and Ni). Metals in each factor are scaled with given factors to the metal of highest abundance for clarity of presentation. Also shown is total ICP-MS metals mass concentration.

Figure S8. Distribution of Pb, Cd, As, Zn, Mn, Tl and Cs with wind patterns at Sarajevo site.

Figure S9. Distribution of Fe, Co, La and Ce with wind patterns at Sarajevo site.

Figure S10. Distribution of Ba and Sr with wind patterns at Sarajevo site.

Figure S11. Distribution of V and Ni with wind patterns at Sarajevo site.

S1. Analyses of PM_{10} filter samples

S1.1 Determination of PM_{10} mass

The gravimetric measurement of PM_{10} samples was carried out before and after the field sampling with an analytical microbalance (Mettler Toledo AX205/A), with a resolution of 10^{-5} g and electrostatic charge outflow systems that eliminate static electricity. The measurement was performed in a balance room where the temperature and relative humidity (RH) were continuously monitored and regulated at a temperature of (20 ± 1) ºC and (45-50)% RH. All of the filters were conditioned for at least 48 h and then weighed for 2 consecutive days.

S1.2 Chemical analyses of PM_{10} filter samples

ETAAS analytical technique was used at the Division of Analytical Chemistry, Department of Chemistry, Faculty of Science, University of Sarajevo, BiH for the determination of cadmium (Cd), copper (Cu), iron (Fe), vanadium (V) and zinc (Zn) in PM_{10} samples. Filter samples were digested with the mixture of HNO_3 (65%), HF (40%) and H_2O_2 (30%)[1]. The concentrations of Cd, Cu, Fe, V and Zn were determined with an electro thermal atomic absorption spectrometer (model AA240Z, Varian, Mulgrave, Australia) equipped with a graphite furnace (GTA 120) and an auto sampler (PSD 120). Each measurement was carried out in 3 replicate measurements. The analytical quality control of sample preparation and ETAAS analysis was checked with a solution of a certified reference material CTA-FFA-1 (fine fly ash, Institute of Nuclear Chemistry and Technology, Department of Analytical Chemistry, Warszawa, Poland), prepared identically as the PM_{10} samples. The element recoveries were in the 80-115% range (Table S1). Standard solutions were prepared daily by diluting the monoelement AAS stock solutions (1000 mg/L). Ultrapure grade reagents were used in the preparation of all samples and standards (Merck, Darmstadt, Germany).

ICP-MS analytical technique was used at the Environmental Hygiene Unit of the Institute for Medical Research and Occupational Health in Zagreb, Croatia for the determination of arsenic (As), barium (Ba), cadmium (Cd), cerium (Ce), cobalt (Co), cesium (Cs), copper (Cu), iron (Fe), lanthanum (La), manganese (Mn), molybdenum (Mo), nickel (Ni), lead (Pb), rubidium (Rb), strontium (Sr), thallium (Tl), vanadium (V) and zinc (Zn) in PM_{10} samples. Filter samples were digested with HNO_3 (65%, Merck, Germany) in a microwave digestion system Ultraclave IV (Milestone Srl, Italy) using an application note for paper filter digestion (maximum temperature 240 °C, maximum pressure 130 bars, and maximum microwave power 1000 W). Selected isotopes ^{75}As, ^{137}Ba, ^{111}Cd, ^{140}Ce, ^{59}Co, ^{133}Cs, ^{65}Cu, ^{56}Fe, ^{139}La, ^{55}Mn, ^{95}Mo, ^{60}Ni, ^{206}Pb, ^{85}Rb, ^{88}Sr, ^{205}Tl, ^{51}V, and ^{66}Zn were analyzed with inductively coupled plasma mass spectrometer (ICP-MS 7500cx Agilent Technologies, Waldbronn, Germany) in collision mode with helium gas for removing the interferences. An internal standard solution containing Sc, Ge, Rh, Lu, and Bi was added to all of the samples. Certified reference materials ERM CZ-120 (Fine dust PM_{10} like, IRMM, Belgium) and NIST 1648a (Urban particulate matter, NIST, USA) were analyzed along with samples for quality control. Recovery rates ranged from 85% to 105% (Table S1). Reagent blanks, laboratory, and field filter blanks were prepared identically as samples and used for contamination control.

In this study, a good correlation was found between the results of the two techniques for the determination of Cd, Cu, Fe, V and Zn at the Sarajevo site, although different sample preparation (i.e., digestion procedure) was used for ICP-MS and ETAAS. ICP-MS results from both sites were used for statistical analysis due to the ICP-MS high sensitivity (i.e., lower DLs), which was particularly important for very low ambient loadings of metals at Ivan Sedlo site.

S2. US EPA BenMAP cost-benefit analysis

US Environmental Protection Agency (US EPA) Environmental Benefits Mapping and Analysis Program - Community Edition (BenMAP–CE) software incorporates databases that include concentration-response relationships, population files, health and economic data needed to quantify impacts of air pollution. A cost-benefit analysis (CBA) was conducted for BiH for the scenario of improved air quality with the reduction of $PM_{2.5}$. For this purpose, we used the BenMAP-CE model with an estimated value of statistical life (VSL) and the health impact function. BenMAP defines VLS as 120 × gross domestic product (GDP) per capita and for BiH this number is \$0.55M. The health impact function (Equation 1) was calculated based on the $PM_{2.5}$ measurements in three major BiH cities (Sarajevo, Tuzla and Zenica) during 2016 (Figures S2-S3) to give an estimate of annual mortality in BiH due to the air pollution (Figure S4). Also calculated was the alternative scenario of $PM_{2.5}$ reduction in 5-95% range, as well as the resulting decrease in annual BiH mortality.

$$\mathbf{\Delta Y = Y_0 * \left(1 - e^{-\beta \Delta PM}\right) * Pop} \qquad \text{Equation 1}$$

where Y_0 is the baseline incidence rate for mortality, ΔPM is the change in $PM_{2.5}$, Pop is the population, and β estimate of the effect was obtained from the epidemiological study of Krewski et al. (2009)[83], as a slope of the dependence line of mass concentrations of $PM_{2.5}$ and the natural mortality logarithm. The cost of improving air quality (Equation 2) was determined by extrapolating the measured $PM_{2.5}$ and applying the Netherlands data to BiH.

$$\mathbf{COST_{BiH} = COST_{ref} * \frac{PPP_{BiH}}{PPP_{ref}} * CNV_{€\rightarrow\$} * INFL * \frac{A_{BiH}}{A_{ref}}} \qquad \text{Equation 2}$$

where PPP is the purchasing power parity, CNV is the currency conversion, INFL is the inflation in 2016 in US dollars (USD), and A is the surface of a country (in this case BiH). Note that custom monetary unit of the BenMAP is USD.

S3. ExternE Health Costs of Energy

Using a top-down approach, we also estimate mortality and morbidity based on energy sector characteristics of BiH. We collect reported electricity usage from 2016 as an example year for the estimated annual premature deaths and respiratory illnesses. ExternE calculates the adverse health effects due to air-pollution as the following:

$$D_{air} = \sum_{0}^{k} M_{low}^{high} * E_{annual,k} \qquad \text{Equation 3}$$

$$R_{air} = \sum_{0}^{k} S_{low}^{high} * E_{annual,k} + C_{low}^{high} * E_{annual,k} \qquad \text{Equation 4}$$

where D_{air} represents air-pollution-related deaths due to air pollution from different energy sources on an annual basis by type, k, ($E_{annual,k}$) for estimated mortality range ($M_{low}^{high} = \frac{deaths}{\text{GWh}}$) and R_{air} represents air-pollution-related respiratory illness due to air pollution from different energy sources on an annual basis by type, k, ($E_{annual,k}$) for estimated serious, S_{low}^{high} , and minor illness range, C_{low}^{high} , per GWh for each energy technology type, k.

The annual energy consumption values (GWh) are applied to the occupational and air-pollution-related risk method called ExternE. The ExternE model predicts health impacts attributable to air pollution and occupational risks for each energy technology expressed per GWh. The data we use for this analysis are from the International Energy Agency (IEA). ExternE accounts for reduction in life expectancy and cancers attributable to exposure to $PM_{2.5}$, sulfur dioxide, nitrogen oxides, and ozone only and does not consider source-specific trace metals in the PM burden. Additionally the top-down approach only relies on known characteristics of lignite, coal, wood, gas, and oil

based on IEA reports. From observational evidence and prior IEA reports, trash burning and waste energy sources comprise a larger amount of energy consumed in BiH, but those sources are neglected from the ExternE analysis. Population density across BiH is assumed to be 60 people/km^2. ExternE also assumes a linear relationship between $PM_{2.5}$ exposure from existing energy sources and premature death.

S4. Enrichment factors analysis

The crustal enrichment factors (EF) were calculated as the ratio of the concentration of measured metal to Fe in each PM_{10} sample divided by the corresponding ratio in soil. Fe was used as reference element for crustal particles, while the surface soil concentrations previously reported by Huremović et al. (2015)[14] and Žero et al. (2017)[1] for the Sarajevo Canton region were used in EF calculation. Difficulties in determining the composition of the crustal source material is the main source of uncertainty in these calculations. Therefore the results are interpreted only qualitatively, where the EF around 1 indicates the crust soil as dominant source and EF > 5 indicates other sources of that metal (anthropogenic or non-crustal). The EF values of the metals measured in this study are shown in Figure S6.

The results show the highest EF values for Cd, As, Zn, Pb and Cu, confirming the previous findings of anthropogenic sources of these elements. The lowest EF values were found for Ce, La and Co which indicates that these elements originate from soil and support the results of factor analysis in this study, which found Factor 2 with Fe, Ce, La and Co as elements derived from crustal source.

Table S1. Recoveries of certified reference material used in this study by ICP-MS (ERM CZ-120 and NIST 1648a) and ETAAS (CTA-FFA-1) analytical techniques

Element	Recovery of certified reference material / %		
	ERM CZ-120 (ICP-MS)	NIST 1648a (ICP-MS)	CTA-FFA-1 (ETAAS)
As	98	102	-
Ba	105	n.a.	-
Cd	98	90	80
Ce	97	83	-
Co	94	85	-
Cs	97	85	-
Cu	89	94	98
Fe	101	92	115
La	95	85	-
Mn	93	94	-
Mo	92	n.a.	-
Ni	98	97	-
Pb	103	99	-
Rb	93	79	-
Sr	98	87	-
V	92	88	81
Zn	100	97	95

Table S2. Overview of sampling sites, periods and analytical techniques used in metals' measurements in this study and other locations presented in Table S3.

Sampling site<br>Location	Type	Sampling period	Method	Reference
**Sarajevo, BiH**<br>**Ivan Sedlo, BiH**	**Urban background**<br>**Rural**	**12/2017 – 02/2018**	**ICP-MS**	**This study**
Sarajevo, BiH	Urban traffic	04/2010-02/2019	ETAAS	Huremović et al. , 2020[21]
Sarajevo, BiH<br>Vogošća, BiH	Urban<br>Rural	06/2013-02/2014	ETAAS	Žero et al., 2017[1]
Belgrade, Serbia	Urban background	09/2007	ICP-MS	Jokšić et al., 2009[47]
Belgrade, Serbia	Urban traffic	07/2003-12/2006	AAS	Mijić et al., 2012[48]
Belgrade, Serbia	Urban traffic<br>Suburban industrial<br>Rural industrial	2011-2015	ICP-MS	Perišić et al., 2017[49]
Bor, Serbia	Urban industrial<br>Urban industrial<br>Suburban industrial	2014 and 2015	GF AAS<br>ICP-MS	Tasić et al., 2017[57]
Zagreb, Croatia	Urban background	2000-2003	AAS	Bešlić et al., 2008[58]
Elbasan, Albania	Urban	Winters 2010-2012	FAAS	Bekteshi and Karamelo, 2017[61]
Budapest, Hungary	Urban traffic	Winters 2004-2007	ICP-SF-MS	Muránszky et al., 2011[55]
Skawina, Poland	Urban industrial	02/2019 – 12/2019<br>02–03/2019 and 10–12/2019	ICP-MS	Zioła and Słaby, 2020[51]
Ziemiecice and Tworog Maly, Poland	Rural background	03/2013 - 08/2013	ICP-MS	Zajusz-Zubek et al., 2015[54]

Table S2. - cont.

Sampling site				
**Location**	**Type**	**Sampling period**	**Method**	**Reference**
Volos, Greece	Seaport	2014 and 2015	ICP-MS	Manoli et al., 2017[52]
Elefsis, Greece	Industrial	Winter 2016<br>Winter 2017	ICP-MS	Koukoulakis et al., 2019[45]
Istanbul, Turkey	Urban	2010	AAS	Aksu, 2015[44]
Salentum peninsula, Italy	Urban background<br>Urban industrial<br>Urban	2003-2010	GF-AAS	Contini et al., 2014[46]
Santander, Spain<br>Maliaño, Spain	Urban background<br>Urban/Industrial<br>Urban	01/2015-01/2016<br>09/2015	ICP-MS	Hernandez-Pellon and Fernandez-Olmo, 2019[56]
Riotinto mining district, Spain	Rural	2009-2014<br>2015<br>2016<br>2017	ICP-MS	Sánchez de la Campa et al., 2020[53]
Alcora, Spain<br>Onda, Spain<br>Castellón, Spain	Urban<br>Urban<br>Urban	2007-2011	ICP-MS	Vicente et al., 2014[60]
Porto – Leça do Balio, Portugal<br>Porto – Vila Nova da Telha, Portugal	Urban<br>Urban	2004-2014	ICP-MS	Albuquerque et al., 2016[59]
Ahvaz, Iran	Urban background	Winter 01-03/2013<br>10/2012 – 09/2013	ICP-OES	Farsani et al., 2018[50]

Table S3. Comparison of PM_{10} metals mass concentrations in this study with other locations. Units of all presented data are ng m^{-3}.

Location	As	Ba	Cd	Ce	Co	Cs	Cu	Fe	La	Mn	Mo	Ni	Pb	Rb	Sr	Tl	V	Zn
**Sarajevo, BiH**	1.98	5.05	0.34	0.17	0.050	0.097	4.41	188	0.081	6.04	0.20	0.69	8.01	2.19	0.96	0.083	0.94	35.1
**Ivan Sedlo, BiH**	0.36	0.54	0.077	0.046	0.022	0.036	0.63	63.0	0.025	2.71	0.034	0.26	2.69	0.36	0.26	0.046	0.29	8.13
Sarajevo, BiH[21]			2.03									16.88	54.83					
Sarajevo, BiH[1]			0.30				36.6	707		17.8		0.57	9.2				3.5	278
Vogošća, BiH[1]			0.30				22.4	186		12.8		0.55	6.7				1.5	294
Belgrade, Serbia[47]	1.026	17.46	0.326		0.392		9.03	1167		12.04	4.56	3.48	74.88			0.04	3.56	53.2
Belgrade, Serbia[48]			1.86				85.1	1407.9		24.5		22.6	47.8				38.3	1221.2
	2.13		0.21							6.03		4.78	8.31					
Belgrade, Serbia[49]	19.11		0.33							8.97		4.35	12.29					
	12.63		0.29							9.75		6.23	12.61					
	37.9		4.2									3.4	289					
Bor, Serbia (2014)[57]	34.1		5.7									3.4	502					
	46.1		9.1									3.2						
	60.7		4.3									4.2	67					
Bor, Serbia (2015)[57]	26.4		2.0									4.0	207					
	109.7		10									3.2						
Zagreb, Croatia[58]			0.76							11.7			85					
Elbasan, Albania[61]			3.29				39.8	8850				91	195					126.3
Budapest, Hungary[55]			1.1		0.24		35	2241		26	2.6	2.3	36	1.7	3.1	0.16	3	74
Skawina, Poland[51]	1.29		0.76		0.78		17.47					2.51	18.1				1.04	52.98
Ziemiecice and Tworog Maly, Poland[54]	8.45		6.35		0.79					2.21		11.88	93.48					

Table S3. -cont.

Location	As	Ba	Cd	Ce	Co	Cs	Cu	Fe	La	Mn	Mo	Ni	Pb	Rb	Sr	Tl	V	Zn
Volos, Greece[52]			30.4					781		20.9		4.88	26					196
	1.71		0.74					874		23.5		5.89	28.7					234
Elefsis, Greece[45]	2.41	9680	0.75		1.04	0.19	7.89	825		15.8		9.12	24	3.93	458	0.34	17	1441
	2.80	9953	0.52		1.79	0.14	15.5	1192		20.1		8.74	7.61	4.18	439	0.06	25.4	2296
Istanbul, Turkey[44]			0.063				0.48	37.22					0.23					0.18
Salentum peninsula, Italy[46]	< 0.9		0.2				9.9	270		6.8		2.7	7.3				1.5	21.8
	< 0.9		0.3				8.5	227.1		3.6		5.2	11.9				1.8	33.6
	< 0.8		< 0.6				11.9	229.2		4.2		2.2	10				< 1.9	23.2
Santander, Spain[56]	0.51		0.45				15.7	242.9		60.8	0.78	0.96	15.6				1.82	103.8
Maliaño, Spain[56]	0.44		1.16				14.4	279.4		231.8	1.03	1.11	6.91				1.12	127.9
	0.38		3.47				8.83	322		721.9	0.83		44.8				1.69	198.6
Riotinto mining district, Spain[53]	1.12	13	0.14	0.62	0.19	0.05	6.1		0.35	8.66	3.27	1.81	4.75	0.95	2.27	0.03	2.78	23.2
	2.98	11.9	0.13	0.66	0.19	0.05	7.65		0.37	70	4.83	1.32	7.2	0.99	2.39	0.03	2.85	21.7
	4.47	24.1	0.15	1.19	0.36	0.09	25.3		0.65	14.1	5.54	1.79	11.5	1.53	3.33	0.06	3.82	49.1
	1.97	12.4	0.23	1.02	0.32	0.08	14.8		0.52	14.7	5.8	3.44	6.12	1.33	2.82	0.03	3.14	21.1
Alcora, Spain[60]			0.20									2.06	0.052					
Onda, Spain[60]			0.19									1.95	0.018					
Castellón, Spain[60]			0.19									2.59	0.026					
Porto – Leça do Balio, Portugal[59]	0.7		0.4				46.9			9.4		3.8	14					341.8
Porto – Vila Nova da Telha, Portugal[59]	0.6		0.4				40.5			7.9		3.2	16.1					128.7
Ahvaz, Iran[50]			0.22		0.85							1.58	5.7					7.95

Table S4. Pearson's correlation matrix between the metal concentrations in PM_{10} samples collected at Sarajevo (N=57). Statistically significant coefficients ($p < 0.001$) are marked red.

	V	Mn	Fe	Co	Ni	Cu	Zn	As	Rb	Sr	Mo	Cd	Cs	Ba	La	Ce	Tl
Mn	0.52																
Fe	0.67	0.87															
Co	0.68	0.87	0.97														
Ni	0.94	0.64	0.78	0.79													
Cu	0.61	0.66	0.74	0.71	0.73												
Zn	0.53	0.85	0.73	0.70	0.64	0.78											
As	0.52	0.74	0.64	0.61	0.62	0.77	0.96										
Rb	0.46	0.61	0.55	0.54	0.59	0.84	0.86	0.90									
Sr	0.16	0.19	0.20	0.25	0.24	0.57	0.28	0.26	0.45								
Mo	0.63	0.59	0.68	0.64	0.71	0.78	0.69	0.69	0.70	0.27							
Cd	0.52	0.77	0.66	0.64	0.63	0.82	0.95	0.95	0.90	0.33	0.71						
Cs	0.45	0.88	0.70	0.68	0.56	0.65	0.91	0.84	0.71	0.20	0.58	0.84					
Ba	0.33	0.35	0.40	0.41	0.44	0.76	0.47	0.46	0.63	0.95	0.45	0.52	0.37				
La	0.38	0.32	0.63	0.65	0.51	0.40	0.14	0.09	0.16	0.17	0.31	0.13	0.10	0.26			
Ce	0.36	0.34	0.64	0.66	0.49	0.41	0.17	0.12	0.19	0.22	0.30	0.16	0.14	0.31	0.98		
Tl	0.27	0.76	0.53	0.50	0.32	0.36	0.68	0.57	0.39	0.04	0.32	0.59	0.90	0.15	-0.06	-0.01	
Pb	0.62	0.79	0.69	0.68	0.69	0.80	0.92	0.90	0.83	0.42	0.70	0.92	0.87	0.59	0.14	0.18	0.67

Table S5. Pearson's correlation matrix between the metal concentrations in PM_{10} samples collected at Ivan Sedlo (N=9). Statistically significant coefficients ($p < 0.001$) are marked red.

	V	Mn	Fe	Co	Ni	Cu	Zn	As	Rb	Sr	Mo	Cd	Cs	Ba	La	Ce	Tl
Mn	-0.15																
Fe	-0.15	0.55															
Co	0.35	0.69	0.61														
Ni	0.86	0.24	0.25	0.75													
Cu	-0.33	0.71	0.13	0.26	-0.20												
Zn	-0.23	0.71	0.00	0.35	-0.05	0.93											
As	-0.30	0.63	-0.06	0.20	-0.18	0.90	0.97										
Rb	0.08	0.60	0.09	0.34	-0.01	0.90	0.83	0.83									
Sr	0.91	-0.25	0.05	0.33	0.83	-0.61	-0.54	-0.61	-0.40								
Mo	0.22	0.30	0.08	0.25	0.16	0.42	0.28	0.32	0.54	0.07							
Cd	-0.26	0.78	0.07	0.36	-0.06	0.91	0.98	0.96	0.77	-0.54	0.33						
Cs	-0.23	0.65	0.02	0.32	-0.06	0.87	0.97	0.98	0.82	-0.53	0.28	0.95					
Ba	0.37	0.52	0.53	0.70	0.68	-0.03	0.06	-0.07	0.10	0.40	-0.22	0.04	0.04				
La	0.35	0.01	0.29	0.49	0.56	-0.58	-0.44	-0.49	-0.41	0.55	-0.02	-0.40	-0.40	0.48			
Ce	0.27	0.08	0.36	0.52	0.53	-0.52	-0.39	-0.45	-0.37	0.48	-0.02	-0.35	-0.35	0.50	0.99		
Tl	-0.18	0.64	-0.01	0.33	-0.03	0.86	0.97	0.98	0.79	-0.48	0.32	0.96	0.99	-0.03	-0.42	-0.38	
Pb	-0.15	0.70	0.06	0.42	0.01	0.93	0.97	0.94	0.87	-0.46	0.48	0.95	0.94	-0.02	-0.40	-0.35	0.96

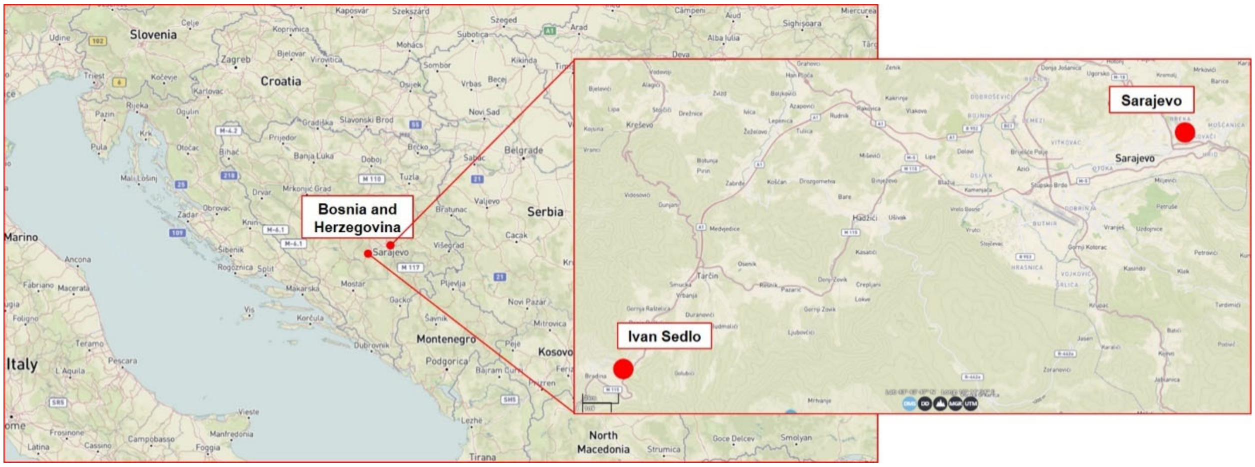

Figure S1. The position of two PM_{10} sampling sites, urban background Bjelave site within the city of Sarajevo and remote Ivan Sedlo site at the mountain ridge 45 km away within the regional (left panel) and local geography (right panel). Created in the US Geological Survey Topographic Maps (https://ngmdb.usgs.gov/topoview/).

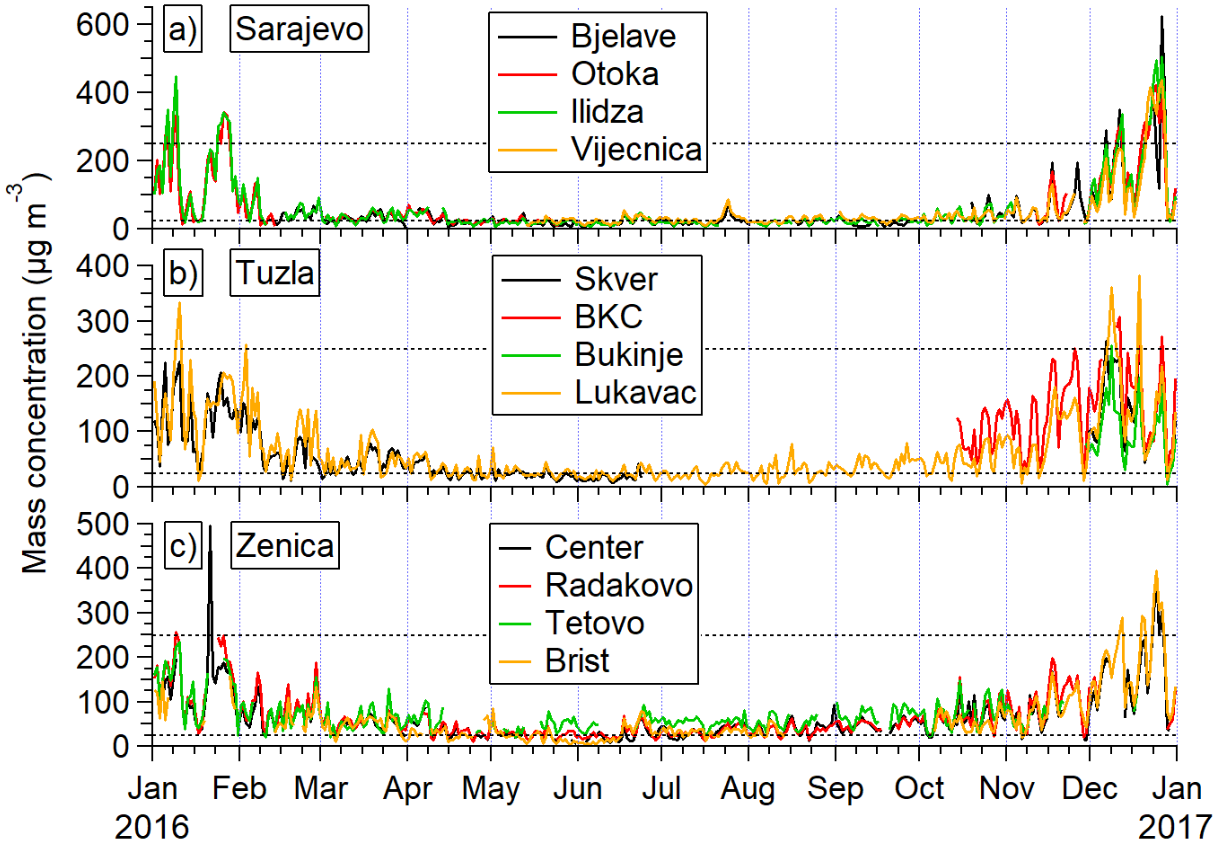

Figure S2. Measured daily average PM mass concentrations during 2016 for (a) Sarajevo (PM_{10}); (b) Tuzla ($PM_{2.5}$); and (c) Zenica (PM_{10}). Lines indicate $PM_{2.5}$ daily recommended value (25 μg/m^3) and the one for hazardous level (250 μg/m3) (EU Directive 2008/50/EC). Names of different measurement sites in each of three cities are noted in each panel. For each town 4 sampling sites were defined, shown in various colors in panels. All data were taken from the Federal Hydrometeorological Institute of Bosnia and Herzegovina, available online[34].

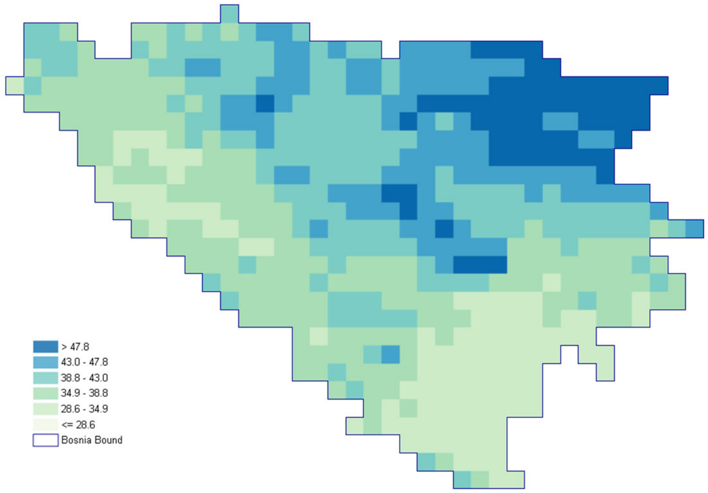

Figure S3. Heatmap of average annual $PM_{2.5}$ mass concentrations in BiH during 2016. Heatmap $PM_{2.5}$ results were produced by BenMAP tool with $PM_{2.5}$ BiH measurements (also shown in Figure S2) as input data.

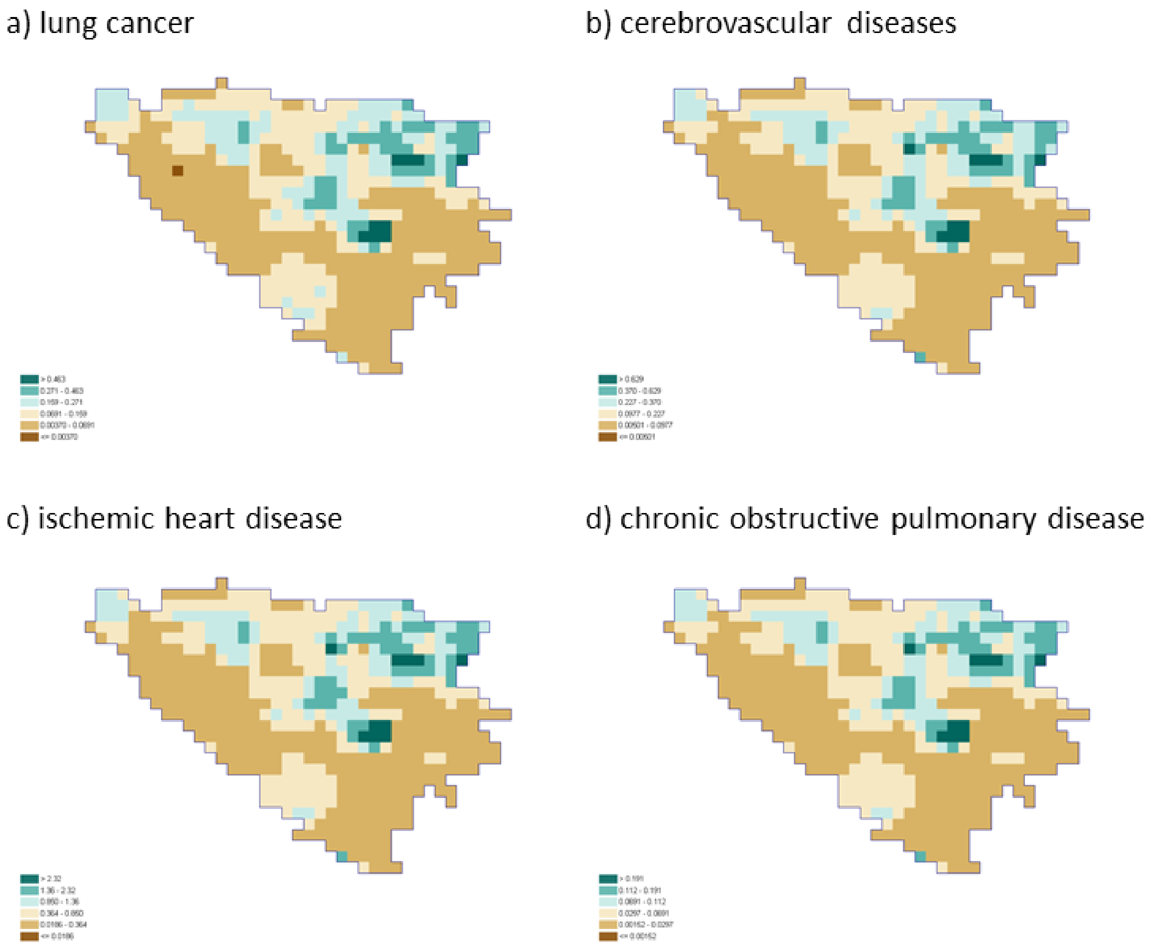

Figure S4. BenMAP results of mortality in BiH from: (a) lung cancer; (b) cerebrovascular diseases; (c) ischemic heart disease; and (d) chronic obstructive pulmonary disease.

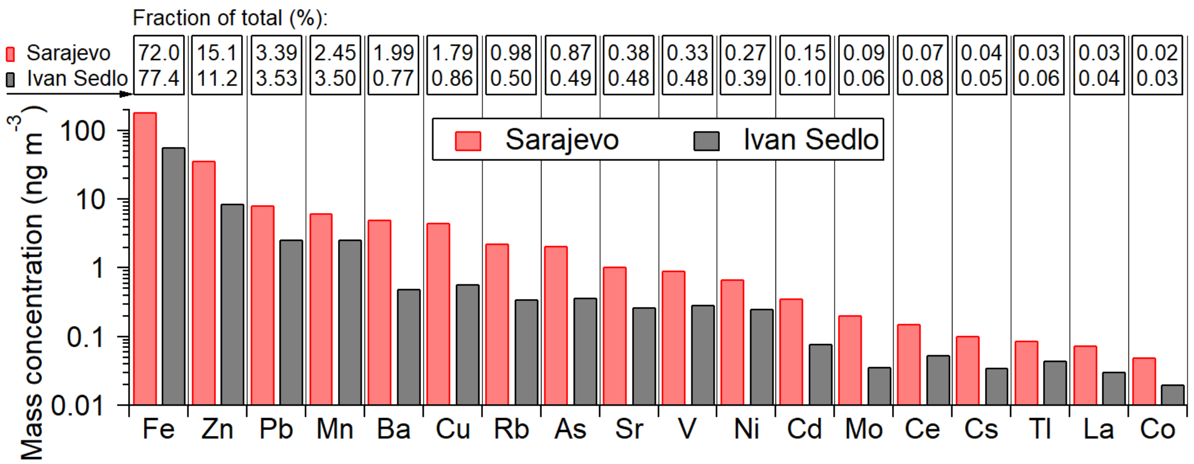

Figure S5. Average mass concentrations of all metals measured by ICP-MS analytical technique at the Sarajevo urban background site and the Ivan Sedlo remote site. Also shown are the percent contributions of each metal to the total ICP-MS metal mass.

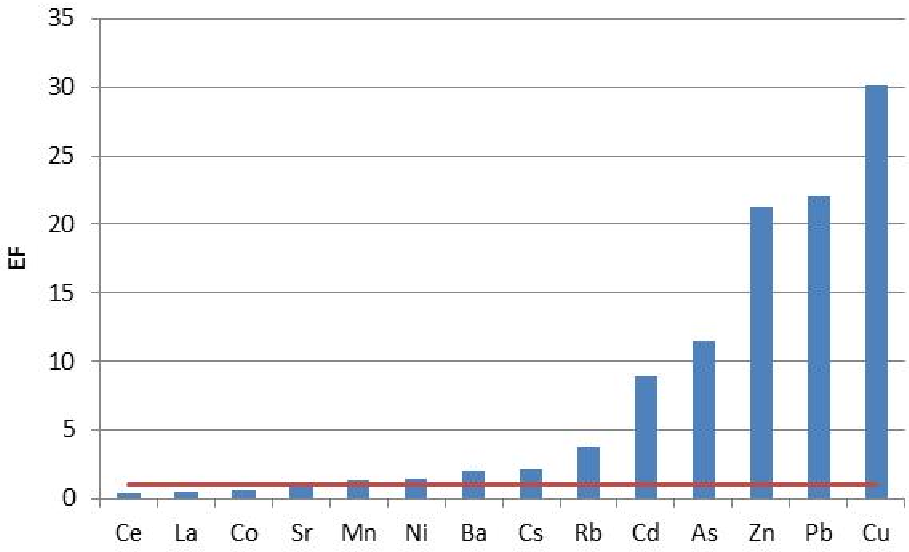

Figure S6. Crustal enrichment factors of metals analyzed in PM_{10} filter samples collected at Sarajevo site (EF=1 is marked as red line).

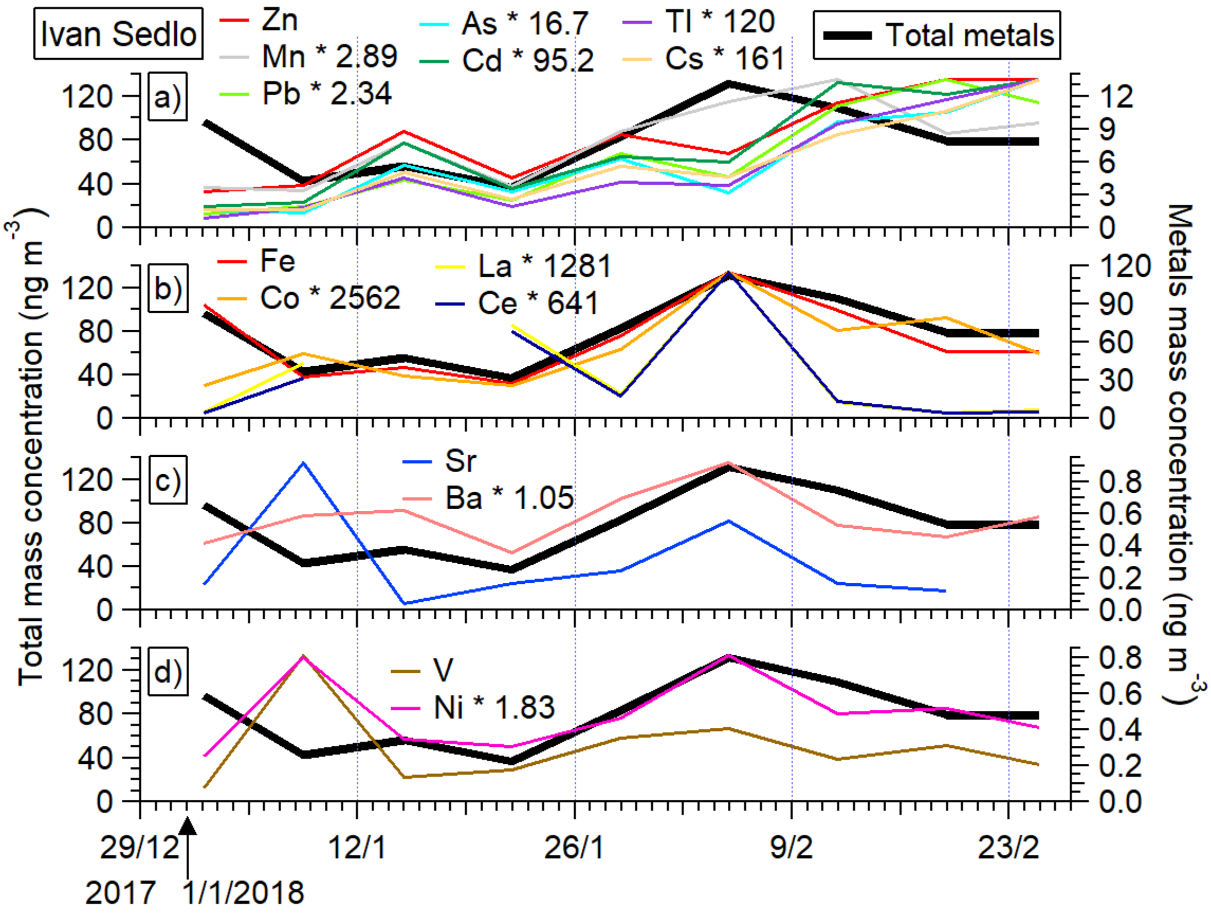

Figure S7. Ivan Sedlo site mass concentrations of metals grouped as the results of factor analysis for Sarajevo site (i.e., PM_{10} metals in each factor): a) Factor 1 (Zn, Mn, Pb, As, Cd, Tl and Cs); b) Factor 2 (Fe, Co, La and Ce); c) Factor 3 (Sr and Ba); and d) Factor 4 (V and Ni). Note that factor analysis was not performed for Ivan Sedlo metals due to a small number of samples (9). Metals in each factor are scaled to the metal of highest abundance for clarity of presentation (scaling factors are also shown). Also shown is total ICP-MS metals mass concentration.

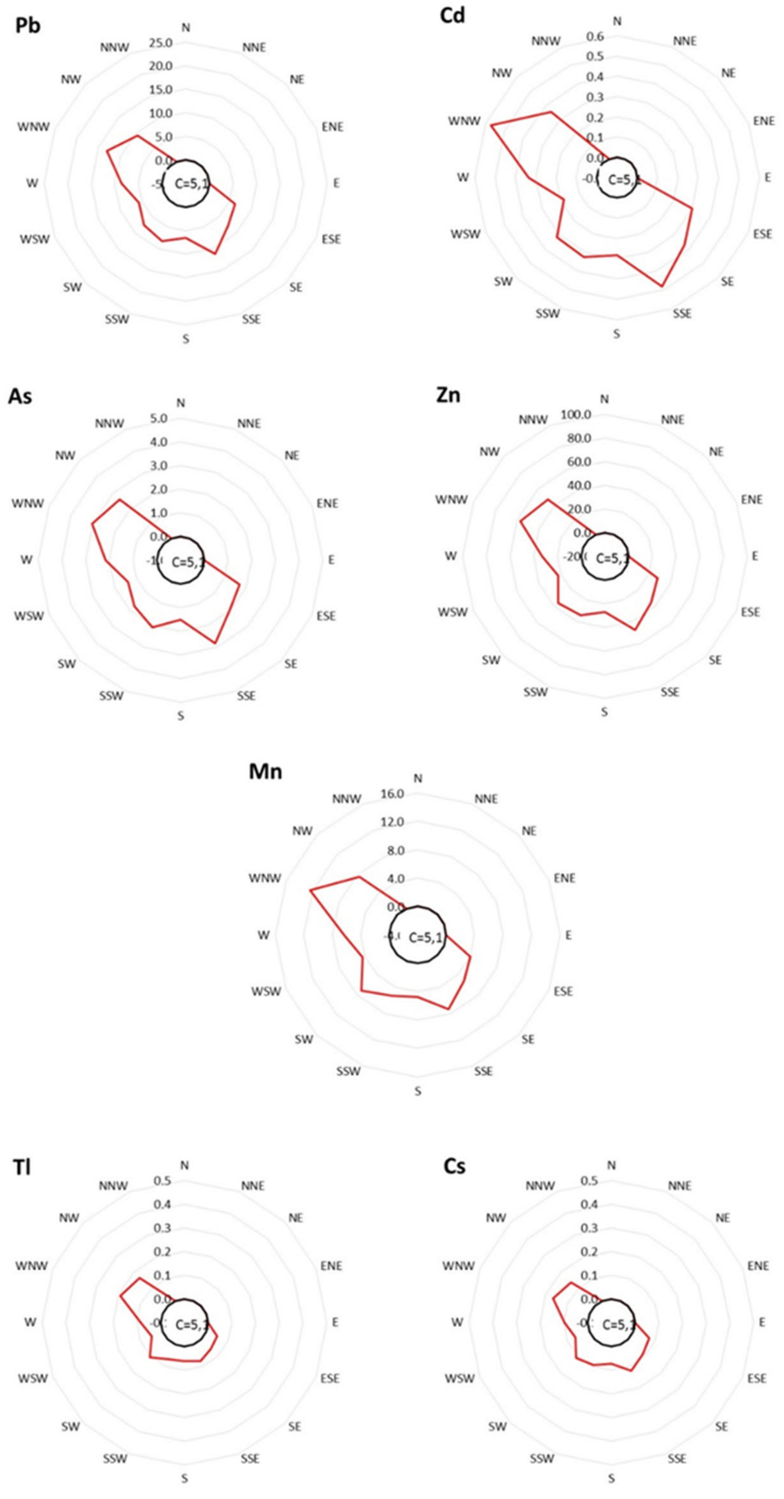

Figure S8. Distribution of Pb, Cd, As, Zn, Mn, Tl and Cs with wind patterns at Sarajevo site.

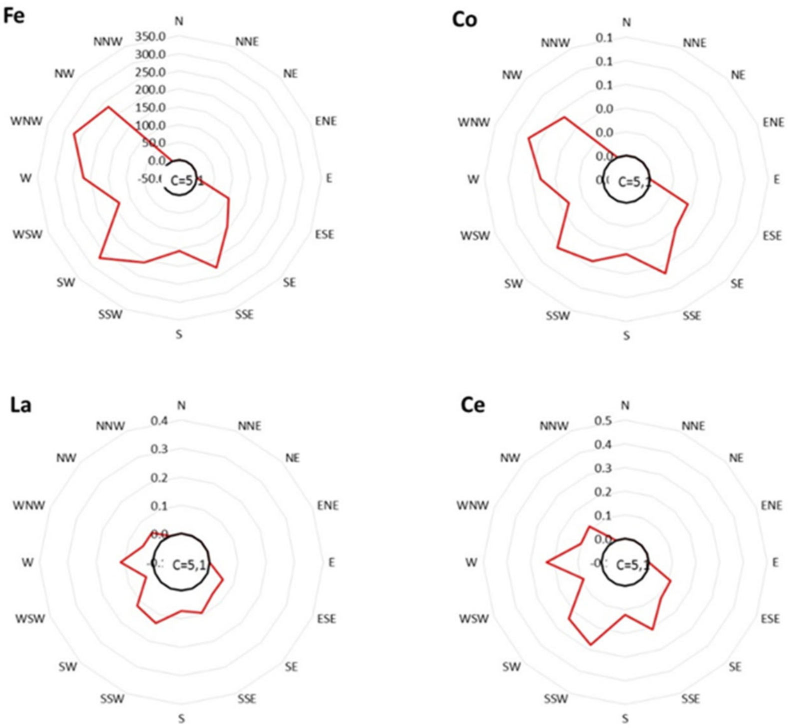

Figure S9. Distribution of Fe, Co, La and Ce with wind patterns at Sarajevo site.

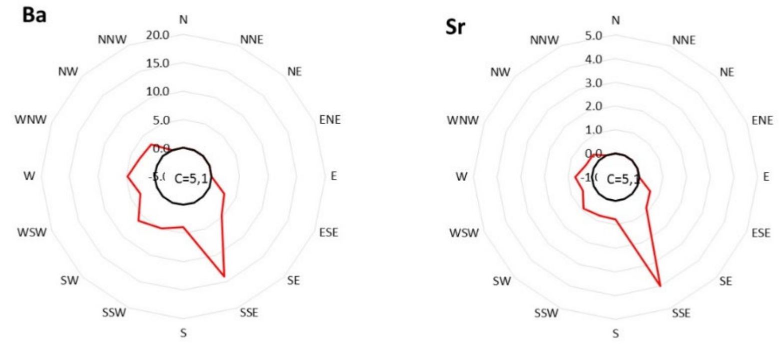

Figure S10. Distribution of Ba and Sr with wind patterns at Sarajevo site.

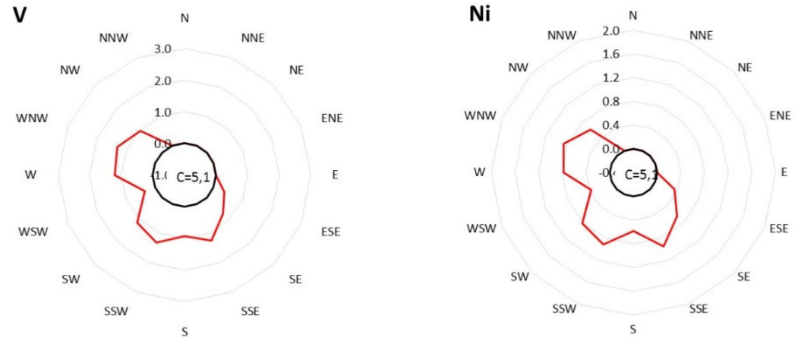

Figure S11. Distribution of V and Ni with wind patterns at Sarajevo site.
